# Biomedical Discovery Acceleration, with Applications to Craniofacial Development

**DOI:** 10.1371/journal.pcbi.1000215

**Published:** 2009-03-27

**Authors:** Sonia M. Leach, Hannah Tipney, Weiguo Feng, William A. Baumgartner, Priyanka Kasliwal, Ronald P. Schuyler, Trevor Williams, Richard A. Spritz, Lawrence Hunter

**Affiliations:** 1 Center for Computational Pharmacology, University of Colorado at Denver, Denver, Colorado, United States of America; 2 Department of Craniofacial Biology, University of Colorado at Denver, Denver, Colorado, United States of America; 3 Human Medical Genetics Program, University of Colorado at Denver, Denver, Colorado, United States of America; University of Tokyo, Japan

## Abstract

The profusion of high-throughput instruments and the explosion of new results in the scientific literature, particularly in molecular biomedicine, is both a blessing and a curse to the bench researcher. Even knowledgeable and experienced scientists can benefit from computational tools that help navigate this vast and rapidly evolving terrain. In this paper, we describe a novel computational approach to this challenge, a knowledge-based system that combines reading, reasoning, and reporting methods to facilitate analysis of experimental data. Reading methods extract information from external resources, either by parsing structured data or using biomedical language processing to extract information from unstructured data, and track knowledge provenance. Reasoning methods enrich the knowledge that results from reading by, for example, noting two genes that are annotated to the same ontology term or database entry. Reasoning is also used to combine all sources into a knowledge network that represents the integration of all sorts of relationships between a pair of genes, and to calculate a combined reliability score. Reporting methods combine the knowledge network with a congruent network constructed from experimental data and visualize the combined network in a tool that facilitates the knowledge-based analysis of that data. An implementation of this approach, called the Hanalyzer, is demonstrated on a large-scale gene expression array dataset relevant to craniofacial development. The use of the tool was critical in the creation of hypotheses regarding the roles of four genes never previously characterized as involved in craniofacial development; each of these hypotheses was validated by further experimental work.

## Author Summary

Recent technology has made it possible to do experiments that show hundreds or even thousands of genes that play a role in a disease or other biological phenomena. Interpreting these experimental results in the light of everything that has ever been published about any of those genes is often overwhelming, and the failure to take advantage of all prior knowledge may impede biomedical research. The computer program described in this paper “reads” the biomedical literature and molecular biology databases, “reasons” about what all that information means to this experiment, and “reports” on its findings in a way that makes digesting all of this information far more efficient than ever before possible. Analysis of a large, complex dataset with this tool led rapidly to the creation of a novel hypothesis about the role of several genes in the development of the tongue, which was then confirmed experimentally.

## Introduction

Human knowledge relevant to biomedical research is expanding at an exponential pace. Over the last twenty years, more than 10 million publications have been indexed by the National Library of Medicine (NLM) and made available through PubMed, reflecting a compounded annual growth rate of more than 4.8% [1],[2]. Structured knowledge, in the form of molecular biology relevant databases, has also been growing at an impressive rate. The journal *Nucleic Acids Research* publishes an annual compendium of peer-reviewed databases relevant to molecular biology; the 2008 issue reported on 1,078 such databases [3].

**Figure 1:**
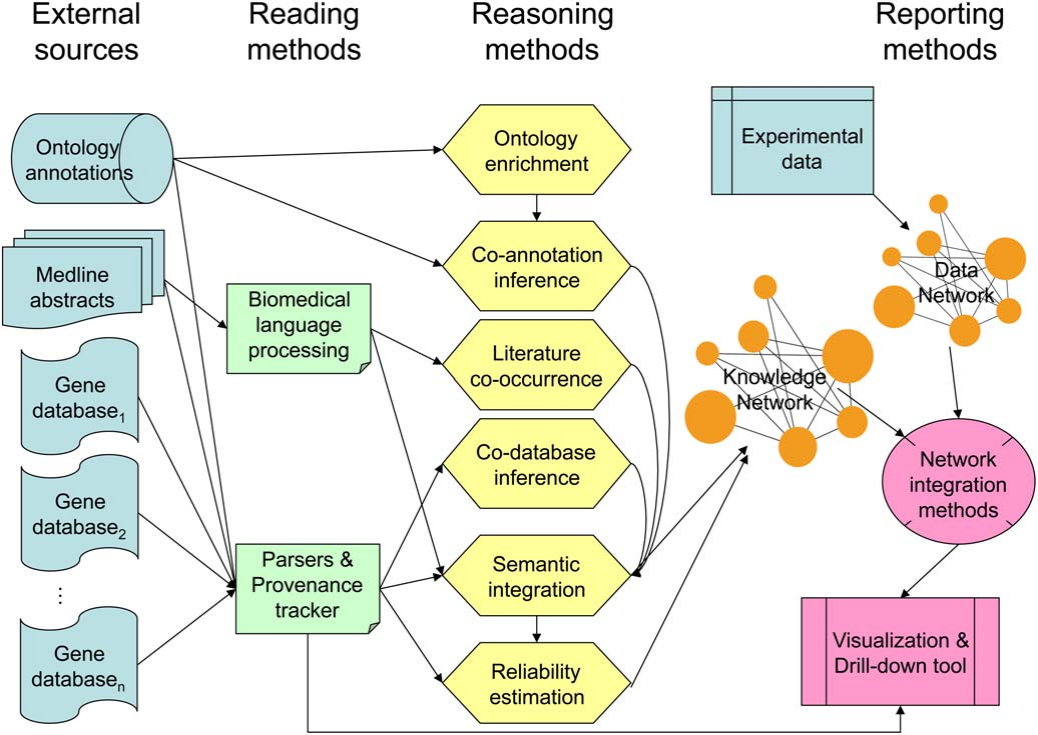
Hanalyzer system diagram. A system diagram describing the modules of the Hanalyzer. Reading methods (green) take external sources of knowledge (blue) and extract information from them, either by parsing structured data or biomedical language processing to extract information from unstructured data. Reading modules are responsible for tracking the provenance of all knowledge. Reasoning methods (yellow) enrich the knowledge that results from reading by, for example, noting two genes that are annotated to the same ontology term or database entry. All knowledge sources, read or reasoned, are assigned a reliability score, and all are combined using that score into a knowledge network (orange) that represents the integration of all sorts of relationship between a pair of genes and a combined reliability score. A data network (also orange) is created from experimental results to be analyzed. The reporting modules (pink) integrate the data and knowledge networks, producing visualizations that can be queried with the associated drill-down tool.

While intense specialization has in many cases made it possible for biomedical researchers to know everything practically relevant in a very narrow domain, a breakdown of disciplinary boundaries and the fundamental interconnectedness of biological systems have rendered specialization an increasingly impractical strategy for keeping up with biomedical knowledge. Information about fundamental molecular structures and functions, such as mutations or protein-protein interactions, are spread across the entire literature. For example, [4; figure 1] demonstrated that nearly 40% of the more than 5,000 journals indexed in PubMed in a typical year contained at least one assertion regarding protein transport, interaction or expression that could be found by a text mining system.

One approach to dealing with this overwhelming amount of information is to organize human experts to curate key aspects of it, resulting in databases of formally represented assertions with pointers to the evidence in the literature. Over the last 6 years, the U.S. National Institutes of Health has invested more than $52 million to support ontology development and use (Personal communication from Peter Good), including the Gene Ontology Consortium and the National Center for Biomedical Ontology. However, even this large investment has been simply unable to keep up with the volume of relevant publications; [1] showed that even under extremely optimistic assumptions it will be decades before annotation will be complete and up to date.

Furthermore, not all human knowledge of biomolecular function is explicitly stated in any database or publication. Molecular biologists often make inferences regarding the likely function of a molecule based on factors such as homology, interaction partners, or other methods; this approach has been called the “post-genomic approach to protein function” [5]. Protein interactions are reasonably well characterized experimentally in yeast, but much less so in other organisms. As of this writing, the Database of Interacting Proteins (DIP)[6] contains records regarding 18,331 interactions among 4,923 yeast proteins derived from 23,344 experiments, likely a close to complete inventory. However, there are only 415 curated interactions among 307 mouse proteins derived from 595 experiments in the database—likely fewer than 1% of the true protein-protein interactions. Recently, computational approaches to protein function inference such as [7],[8],[9] and others have extended interaction predictions to generate functional categorization of dramatically larger numbers of proteins. As these inferences of function are less reliable than experimental observations, most computational approaches associate a likelihood or reliability with each prediction.

Advances in instrumentation are also generating molecular data at ever increasing rates. High-throughput (also known as genome-scale) assays for detection and analysis of gene expression, genetic polymorphisms, macromolecular interactions and other fundamental processes are generating datasets that contain information about the structures or activities of on the order of 10^6^ different genes or gene products at a time. More than 200,000 such assays from more than 8,300 different experiments are publicly available from the National Library of Medicine's Gene Expression Omnibus catalog (http://www.ncbi.nlm.nih.gov/geo/ viewed on April 7, 2008), and far more results of high-throughput experiments are available in more restricted settings.

Experiments that exploit these genome scale assays often generate results that implicate dozens to hundreds of genes or gene products related to a phenomenon under study. The amount of information regarding even just these significant results (and relevant homologs) in gene-centric databases and in the research literature is often overwhelming, yet the proper interpretation of the results requires taking stock of all of that knowledge. Furthermore, as the revolution in systems biology has made clear, it is critical to analyze the specific interactions among the genes, not just the genes in isolation. In a set of hundreds of relevant genes, there are tens of thousands of potential interactions to consider. Analyzing all of the relevant genes and interactions in genome-scale data, while important to advancing human understanding of biomedical phenomena, is a truly daunting task.

## Methods

Here we introduce a novel computational approach to analyzing genome-scale data in the light of existing knowledge, built on three broad classes of algorithms: reading, reasoning and reporting. For that reason, we refer to the overall approach as a 3R system. 3R systems are a restricted class of knowledge-based systems. The goal of a 3R system is to assist biologists in forming *explanations* of the phenomena in genome-scale data, and to generate significant hypotheses that can influence the design of future experiments. The approach is based on the comparison of two weighted graphs. One graph, called the “knowledge network,” represents a large portion of the existing knowledge of gene products and their relationships. The other, a “data network,” describes a particular data set produced by a high-throughput experiment. There are many possible ways to implement a 3R system; we call the particular implementation reported on here the Hanalyzer (for high-throughput analyzer).

This paper describes the use of the Hanalyzer in the analysis of a comprehensive expression dataset for mouse craniofacial development. (This dataset is described in detail in [10]; the analysis of a portion of the data not reported by [10] is described below.) The Hanalyzer does not automate the production of explanations (nor hypotheses), but supports human users who are performing these tasks. Through use of the Hanalyzer, several novel hypotheses regarding the gene networks involved in craniofacial biology were generated; we also report on their experimental validation.

A wide variety of previously reported systems and algorithms have influenced this work. The descriptions of the reading, reasoning and reporting components below cite related work and compare specific approaches. With respect to the overall system architecture, there is substantially less related work. Many reported uses of background knowledge in the analysis of high throughput data use it as the basis for clustering differentially expressed genes or to attempt to model pathways or networks; such work is reviewed in [11]. Other approaches use background knowledge to identify *a priori* sets of related genes for differential expression testing, e.g.[12] or [13]. [14] describes InteractionFetcher and CytoTalk, two Cytoscape plugins that facilitate lookups of information about genes in an interaction graph and can assert new edges based on interaction information from remote databases; they describe a use-case analyzing Hepatitis C with their tools. Perhaps the closest previous approach is the case study described in [15], where a protein-protein interaction network was built using the MedScan text mining approach [16] and then applied to analysis of expression array data with the active subnetwork algorithm [17].

The 3R approach differs from this prior work in several ways. First, the use case of developing explanations for the data, rather than identifying or clustering differentially expressed genes, influences both the methods employed and, most importantly, the criteria used to evaluate such a system. Second, while the aforementioned systems are all designed for the specific analysis of gene expression array data, 3R systems can be applied to many other forms of high throughput data, as described in the discussion. Third, our representational commitment to nodes as fiducials both expands and constrains the sorts of knowledge graphs that can be produced and applied. Finally, our division of the approach into reading, reasoning and reporting tasks expands the sorts of algorithms that can be productively applied to improving performance of 3R systems; reasoning (in the Hanalyzer, the network inference algorithms) in particular had not previously been applied in this sort of analysis.

As shown in the system diagram in Figure 1, 3R systems involve reading, reasoning and reporting. The reading component extracts information from the literature and from relevant databases. The reasoning component makes inferences regarding several types of semantic relationships among genes and gene products, estimating likelihoods and leaving a trail of provenance. The reporting component relates knowledge to data and presents the combinations by augmenting a popular visual interface. Underlying each of these tasks is a shared knowledge representation capable of supporting the required inference and record keeping.

### Knowledge representation formalism

Knowledge in our system is constrained to be compatible with the World Wide Web Consortium's Web Ontology Language (OWL). While program internals represent the knowledge graphs more directly, it is always possible for the Hanalyzer to export an OWL version of its knowledge, and to import knowledge in OWL format. The OWL syntax for representing properties is a binary relation, linking two individuals or an individual and a value; however, for a great deal of knowledge in molecular biology it is natural and convenient to link an individual to more than one other individual or value – for example, the process of transporting a protein from one subcellular location to another would naturally involve a relation between the protein and two locations. For this reason, we adopt the practice recommended in the W3C working group note *Defining N-ary Relations on the Semantic Web* of 12 April 2006 (http://www.w3.org/TR/2006/NOTE-swbp-n-aryRelations-20060412/) pattern 1, primarily following use case 3. The quantification links (described in more detail below) follow use case 1. Provenance information is stored as an annotation property.

Entities in our knowledge network that are clearly and unambiguously interpretable by the biomedical community are termed “fiducials.” A fiducial is either a specific element of a community-curated ontology (such as available through the National Center for Biomedical Ontology's BioPortal, http://www.bioontology.org/bioportal.html) or derived from a specific entry in a publicly available database, such as a particular identifier from the Entrez Gene database (http://www.ncbi.nlm.nih.gov/sites/entrez?db=gene). All representations of genes, gene products, macromolecular sequence features, molecular functions, biological processes, metabolic pathways, subcellular locations, cell types, organisms, diseases and drugs in our system are fiducials.

Not all relationships between entities in our knowledge network can be mapped to elements of a community curated ontology. Arcs between fiducials are used to represent non-fiducial elements. For example, in addition to relations defined in the OBO Relation Ontology (http://www.obofoundry.org/ro/) such as ‘part-of,’ we use at least two additional relationships: One non-fiducial link represents the very abstract relationship that specifies a connection of any kind between a pair of proteins. Ultimately, the user interface displays this very abstract relationship, which, borrowing terminology from [9], we will refer to as a *semantic relationship*. The other non-fiducial link quantifies the overall inferred reliability of the semantic relation (see below for how this is calculated). Also, as described in detail below, some knowledge sources may assert links among fiducials that do not correspond to relations from the OBO Relation Ontology. The totality of all entities and relationships in this knowledge representation in the system at any given time is called the *knowledge network*.

### Populating an initial knowledge network by reading

The fiducials and semantic relationships in the knowledge network are initially populated by a series of processes that extract information from databases and from the literature. Again following the terminology of [9], we call these processes “experts.” The size of the graph produced can be limited by seeding the knowledge-base with a target set of fiducials (usually a set of genes of interest from a particular experiment), and requiring any addition to the knowledge-base to have a relationship involving one of these target fiducials. Unless otherwise noted, the knowledge networks discussed below begin from a target list of 8923 *Mus musculus* genes that were differentially expressed among at least one pair of conditions in the craniofacial dataset described below. The genes were specified by identifiers from the Mouse Genome Informatics (MGI) database [18] (or Entrez Gene or Uniprot IDs, which can be readily translated), and no distinction is made between genes and gene products.

Relationships describing protein-protein interactions are extracted from the Biomolecular Interaction Network Database (BIND) [19], Database of Interacting Proteins (DIP) [20], Molecular Interaction database (MINT) [21], the IntAct database [22] and the RIKEN protein interaction table [23]. Additional relationships are taken from the list compiled by [24] which relates a protein annotated to the Gene Ontology [25] Molecular Function term “protein binding” (GO:0005515) with evidence code IPI (inferred from physical interaction) to the protein identified in the “with” field of the term annotation. Interactions from all databases are combined and divided into experimental assay groups by canonicalizing spelling variants among text strings describing the assay (e.g., TAP and tandem affinity precipitation) and grouping like assays (e.g., CLASSICAL-TWO-HYBRID, MATRIX-TWO-HYBRID, TWO-HYBRID, TWO-HYBRID-ARRAY, TWO-HYBRID-TEST all represented by the single label TWO-HYBRID). Any relationship without an assay description is labeled UNKNOWN. This process results in 4,544 relationships among 1,693 targeted MGI identifiers with 25 assay type labels. Each assay type becomes an “expert,” and can therefore be assigned a reliability score independent of the other assays.

Relationships describing protein-DNA interactions are extracted from the TRANSFAC 10.2 database [26] by relating a protein to the transcription factor recognizing a given sequence motif found in the regulatory region of the protein. The expert derived from this information (Transfac) contains 580 relationships among 434 MGI identifiers. Additional putative protein-DNA interactions are extracted from the PReMod database of genome-wide mammalian cis-regulatory module predictions [27] which catalogs phylogenetically conserved regulatory modules between human and mouse. The resource lists the TRANSFAC motif identifiers of elements in a conserved module, together with the upstream and downstream genes. Two different experts are derived from this information, one which relates a transcription factor recognizing any motif in the module to both the upstream and downstream genes (PReMod) and one which relates two transcription factors if they recognize motifs in the same identified conserved module (PReMod_M_). The PReMod expert asserts 345,814 relationships among 13,852 targeted MGI identifiers while the PReMod_M_ expert asserts 17,317 relationships among 189 targeted MGI identifiers. The large number of relationships from these experts suggests the potential of a high level of noise, which is expected for computational predictions.

The OpenDMAP system [4] was used to extract information from all abstracts in Medline regarding protein transport events, protein-protein interaction assertions, and what proteins are expressed in which cell types. OpenDMAP is particularly well suited to this task, since its information extraction patterns are explicitly associated with a knowledge-base, and all of its outputs are in terms of the representation scheme of the knowledge-base. Although discussed in detail in [4] a brief example describing the extraction of protein transport assertions from the literature here is illustrative. Protein transport is a 4-place relationship between two proteins (a transporter and a transportee, represented by MGI IDs) and two subcellular locations (fiducials from the Gene Ontology cellular component subtree). Most assertions do not mention all aspects of that relationship, although to be extracted at least one protein and one compartment had to be recognized. To map this extracted information into the network, up to five pairwise relationships are created. An expert (Transloc) derived from this information asserts a relationship between the transporter and the transportee, using the Entrez gene ID to MGI identifier mapping available at the MGI website, for a total of 157,764 interactions among 1108 targeted MGI identifiers.

Protein-protein interactions extracted from the literature can be translated into network arcs straightforwardly. Extracted assertions regarding the type of cell that a protein was expressed in were mapped to a relation between a gene and an element of the cell type ontology. A total of 265,795 interaction instances and 176,153 expression instances were extracted from all Medline abstracts. Of these, 8292 interaction instances and 7035 expression in cell type instances could be mapped to a targeted MGI mouse gene, resulting in the assertion of 4525 relations among 3157 genes based on literature assertions of protein-protein interactions, and 127,283 relations among 1677 genes being expressed in the same cell type (fiducials from the Cell Type Ontology).

Even when the previously described information extraction system is unable to extract a direct relationship from biological literature, systematic overlap between publications that merely mention two genes can be taken as indirect evidence of a semantic relationship between them. Several systems have used the existence of an article that mentions a pair of genes as evidence of an interaction between them (e.g. [28]). Others use a probabilistic measure based on mutual information [29] or the hypergeometric distribution [30],[31] and extract relationships exceeding a probability threshold. However, [32] demonstrates that a related measure (thresholded asymmetric co-occurrence fraction or ACF) provides more robust performance in network-based protein function prediction. Since the reliability of finding and normalizing gene mentions in free text is substantially higher than that of more general information extraction [33], we apply this technique in addition to the OpenDMAP approach described above. The ACF measure [32] calculates the proportion of the number of shared mentions relative to the number of mentions the less frequently-mentioned gene in a given pair, incorporating a bias toward relationships involving less well-studied genes. The result is a set of inferred relationships between a pair of genes whenever their ACF>0.5; this expert is called co-Lit.

Each expert also records the support for each assertion it makes, including at least a pointer to the source of the data and, when possible, a publication (as a PubMed identifier) and the date when the assertion was created. The reporting component can show this provenance information and link to the original database entry or document passage during analysis.

Note that finding multiple relationships between a single pair of entities is entirely possible. For example, a pair of proteins may be related via an expert that extracts knowledge from a protein-protein interaction database, and by another that does text-mining searches for protein transport statements in Medline abstracts. When multiple relationships are found between a single pair of entities, the reliability of the semantic relationship increases.

### Inferring additional relationships through reasoning

Once the initial knowledge-base is created, it is enhanced by reasoning processes that add additional relationships. These processes are also called *experts*. When necessary for clarity, experts that obtain knowledge by reading an external source are called reading experts, and those that infer additional knowledge are called reasoning experts.

An important method for adding semantic relations between genes is to infer that such a relationship exists when two genes have certain properties in common. A series of experts asserts semantic relationships between pairs of genes based on: shared membership in a signaling or metabolic pathway (co-KEGG) [34], shared annotation to a particular biological process (co-BP), molecular function (co-MF) or cellular component (co-CC) [25], shared gene knockout phenotype (co-Pheno) [18] or shared protein domain assignment (co-Interpro) [18]. For resources involving a nested hierarchy of ontology terms, such as the Gene Ontology (GO) or the Mammalian Phenotype (MP) ontology, relationships exist at a number of levels. For MP, a relationship is added among proteins annotated to their most specific term while for GO, certain terms are first merged when the information content score by the Jiang measure between the terms exceeds 19.0 (see [35],[36] for details). These experts assert between 7,873 (co-KEGG) and 267,317 (co-BP) relationships covering a combined total of 22,922 MGI identifiers.

Another set of inferred relationships links sets of ontology terms using the ontology enrichment process described in [37] to link molecular functions and biological processes from the Gene Ontology to small molecule participants from the Chemical Entities of Biomedical Interest (ChEBI) ontology. For example, this process creates relationships between the GO molecular function terms “Calcium Signaling” and “Calcium Transport” and the ChEBI term “Calcium(2+).” Additional semantic relationships between genes are inferred if such enrichment results in two genes sharing a small molecule participant in a molecular function or biological process (co-ChEBI). For example, this inference adds a semantic relationship between pairs of genes that have functions each of which in turn has calcium as a participant. Similar inference is made over the GO cross-products (see [38] and http://wiki.geneontology.org/index.php/Cross_Product_Guide).

### Estimating the likelihood of a semantic relationship

A critical aspect of the reasoning component is the ability to assimilate the information from all experts and estimate the confidence that a relationship exists between any given pair of proteins. The collection of assertions from both reading and reasoning experts contains a large number of false positives due to uncertainty in a computational prediction, experimental noise in an assay, or even the intentionally noisy nature of inferred relationships. For example, it is not likely that all cytoplasmic proteins interact as the co-CC expert suggests, yet co-localization information can usefully contribute to estimating the likelihood of a semantic relationship when integrated with the other evidence types.

Biological data integration techniques have been widely studied in the literature, ranging from simple measures which assign higher confidence to assertions shared by multiple experts [39],[40] or based on certain relationship network topology characteristics [41],[42],[43],[44], to more sophisticated integration strategies which use machine learning techniques to estimate interaction likelihoods, such as probabilistic graphical models [45],[46],[47],[48],[49],[50],[51],[52],[53],[54],[55] or kernel methods [56],[57],[58]. Many of the techniques attempt to estimate error rates of the individual expert types before integration using either a gold standard [59],[60],[61],[62],[63],[64],[65],[66],[67],[68], or the set of data sources themselves to determine relative reliabilities [36],[69].

In mouse, there are already too few available sources for determining relationships to justify withholding one as the gold standard. Moreover, since our system attempts to capture a variety of semantics for what type of relationship might exist between two entities, determining the appropriate gold standard is difficult. The consensus reliability estimate [36] used in our system avoids the use of an explicit gold standard by computing the consensus number of assertions for a given relationship among all experts and assigning a higher reliability to a given expert if many other experts agree with its assertions on average (see [36] for details). Since many of the reasoning experts assert a large percentage of all possible relationship pairs, the consensus numbers used in the averaging are computed only over experts which are derived from sources explicitly naming both proteins (protein-protein interactions, 25 experts), protein-DNA interactions (Transfac, PReMod, PReMod_M_), translocation events (Transloc), and literature co-occurrence (co-Lit)). All assertions from a given expert are assigned the reliability of that expert.

One of the most popular methods to combine individual reliabilities is to assume independence of experts (naive Bayes assumption) and compute the integrated likelihood *P* for each relationship using the Noisy-OR function *P = 1−Π_i_ (1−r_i_)* where *r_i_* is the reliability of an expert *i* (scaled if necessary into the range 0 to 1 to allow interpretation as probabilities) [53],[65],[66],[67], see also the useful exposition [70]. The Noisy-OR function has the useful property that the probability of a relationship is high with at least one reliable assertion yet increases with additional support. This property is especially relevant in biology, where it is often difficult to identify false negatives; a given assertion is strengthened by additional information but unlike the case for estimating the reliability of an expert on the whole, an individual assertion is not penalized for lack of additional evidence. Moreover, since the experts are assumed to be independent, experts can be removed from or added to the analysis without excessive re-computation.

### Reporting: Analyzing data using a knowledge network

The purpose of building this large, integrated network is to facilitate the exploration of high-throughput data in light of what is already known, with the goal of generating explanations of the observations and also generating novel biological hypotheses. In order to report the aspects of the knowledge network that are relevant to understanding a dataset, it is necessary to both select appropriate sub-networks for presentation and to present them in a comprehensible and useful way.

A simple approach involves visualizing the knowledge network that includes particular fiducials. A gene list can be used to generate the knowledge sub-network that includes them, similar to the approaches presented in [47],[71],[72],[73]. Such a sub-network can also be extended to include other genes linked to the query set by sharing highly interconnected subcomponents that might represent protein complexes (see for example [44],[74].

**Figure 2:**
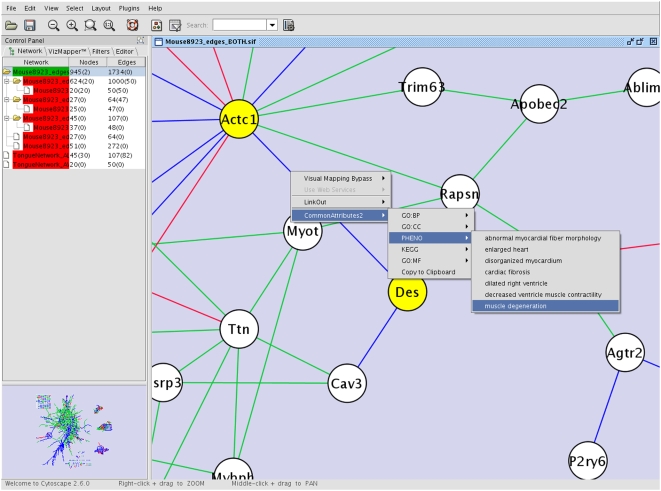
Visualisation of knowledge network via Cytoscape *CommonAttributes* plugin. Screenshot illustrating the use of the *CommonAtrributes* plugin developed to aid exploration of the knowledge network within Cytoscape. Here the linkage between two genes, *Des* and *Actc1* (yellow filled circles), is being explored. By right-clicking on the edge between these two genes, a drop down menu appears including the CommonAttributes2 label which points to the five experts (GO:BP, GO:CC, PHENO, KEGG and GO:MF) which support linking *Des* and *Actc1*. By selecting one of these experts, the attributes common to both genes from that expert are revealed. In this instance, it can be seen that *Des* and *Actc1* share seven phenotypic traits when knocked out or perturbed in mouse models.

Sub-networks, along with attributes of the fiducials and linkages among them are visualized using Cytoscape [75], an open source network visualization platform. A Cytoscape plugin, *CommonAttributes*, was written that allows the user to trace the provenance of links in the knowledge network and directly access the underlying data sources and publications (Figure 2). This approach was used to identify functional explanations of a gene list by exploiting inferences not available in any of the individual data sources [76].

A more effective method of exploiting the knowledge network is to create another quantitative network based on the experimental data (called a data network) and combine the two networks in various ways. In the application reported here, a data network is constructed from the results of a gene expression array experiment. Nodes in this data network are genes that exhibited differential expression in the experiment, arcs connect genes whose expression levels are correlated at an above threshold level, and arc weights are the absolute value of the correlation coefficient (see below for details). Data networks can be generated from any sort of data that can be represented as a weighted graph among fiducials, not just expression arrays. Methods for combining the knowledge network with the data network can highlight linkages in the data that are well supported by existing knowledge, thereby facilitating explanation, or can highlight linkages in the data that are not well supported by existing knowledge, facilitating the generation of novel hypotheses. Both approaches can be exploited together, as demonstrated below.

Other studies have used protein interaction networks together with p-values from tests of differential expression gene expression to identify ‘active’ sub-networks in an input graph [17],[57],[77],[78] and to improve expression profile clustering using a combined distance metric computed from profile correlation and network distance [79],[80]. Our system integrates these two prior approaches, using a combined distance metric to identify active (and explanatorily interesting) sub-networks.

The distinction between the knowledge network and the data network allows comparison at the level of networks, in the spirit of multiple graph approaches such as [81],[82]. Our approach builds on those in two ways: by exploiting the inferences in a dynamically generated and extremely broad knowledge network, and by offering multiple combination functions that support both explanation and hypothesis generation applications.

### Integrating networks through combination functions

Creation of combined networks that integrate the knowledge and data networks in different ways is a key step. Due to the use of fiducials in both the data and knowledge networks, aligning the nodes of these networks is trivial. In contrast, there are many alternatives for combining the arc weights from the knowledge and data networks.

The semantic integration combination functions (e.g. noisy-OR) could also be used to combine corresponding arcs in the data and knowledge networks, but many other alternatives are also available, and some are superior. Approaches based on likelihood ratios for individual sources [60],[64],[83],[84] typically assume independence (naive Bayes) and simply multiply likelihoods. When the assertion probabilities can be interpreted as p-values, [69] review three techniques from statistical mechanics for integration: Fisher's F, Mudholkar-George's T, and Liptak-Stouffer's Z (see [85]). Averaging the probabilities or averaging logistic functions of the probabilities, as used in [80] are also possibilities.

**Figure 3:**
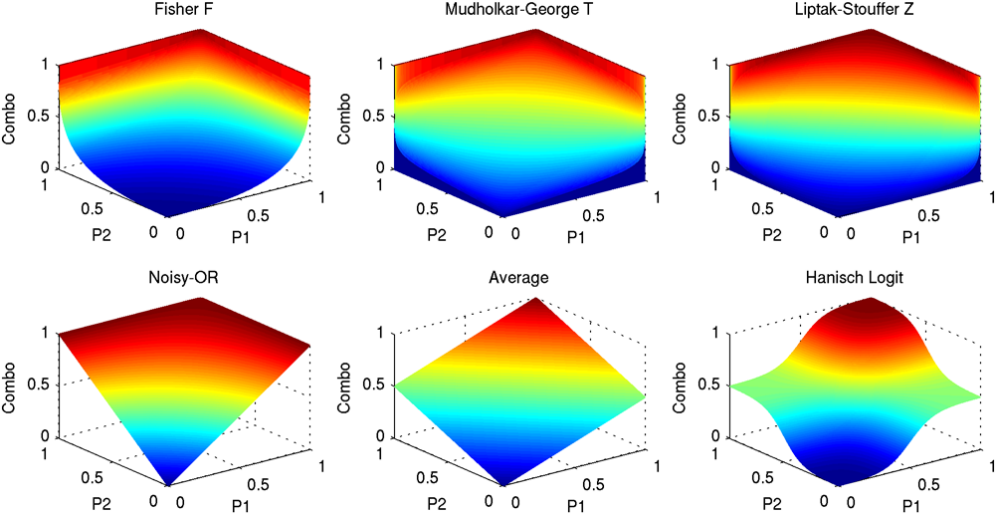
Comparison of probability combination functions. The choice of probability combination functions in semantic integration and in the combination of knowledge and data networks is critical to the utility of the system. This figure shows the global characteristics of a variety of possible combination functions. Probabilities from two sources P1 and P2 (horizontal plane) are combined. Color indicates the magnitude of the combination (vertical axis) from 0.0 (blue) to 1.0 (red). Application of Fisher's F, Mudholkar-George's T and Liptak-Stouffer's Z has been modified their treatment in [69] to emphasize agreement on high probabilities rather than low p-values. The s and v parameters of the logistic function (Logit) were estimated as in [80]. The probability of a network edge given by the knowledgebase is calculated as described above, using the Noisy-OR function with the CONS reliability *P_net_ = 1−Π_i_ (1−r_i_)*. The probability from the external expression data source for an edge between two proteins *x* and *y* is simply the absolute value of the Pearson correlation coefficient computed between the expression profile vectors *P_exp_ = | correlation(x,y) |*. The edge probabilities from the two sources are then combined either using the average of the probabilities Average* = [P_net_+P_exp_]/2* or the average of logistic functions of the probabilities Logit* = [logistic(P_net_)+logistic(P_exp_)]/2* where *logistic(X) = 1/(1−e^−s(X−v)^)*. As in [80], the parameter *v* is set to the mean of the corresponding distribution and the parameter *s* is set to *6/v* to yield a moderate slope. The reporting component of the system then uses the values of the combined function to extract sub-networks of high probability, either by including all edges exceeding a given score or the set of top scoring edges.

The effect of combining probabilities from two sources using various techniques is illustrated in Figure 3. All functions except those denoted Average and Hanisch Logit exhibit the behavior described earlier about Noisy-OR, where the value of the combined probability is 1.0 if at least one of the sources assigns a probability of 1.0 (observed as the red area touching the z = 1.0 plane). Mudholkar-George's T and Liptak-Stouffer's Z have the additional property that the combined probability is 0.0 if at least one of the sources assigns a probability of 0.0 (observed as the dark blue area touching the z = 0.0 plane). In this context, these two functions are less applicable since negative relationships (probability of 0.0) are difficult to observe. The remaining functions differ on how they treat intermediate probability values. Fisher's F shows a rapid decline in combined probability compared to Noisy-OR which maintains a higher combined value when at least one is high. In contrast, Averaging and Hanisch Logit methods require agreement among sources to achieve a high combined value, allowing a value of 1.0 only when both source probabilities are 1.0. The sinusoidal curve of Hanisch Logit implements a thresholding effect where the combination is given more weight than in Averaging when both source probabilities are at least greater than 0.5. Since the purpose of the combination network is to emphasize concurrence among the knowledge and the data networks, the Averaging and Logit methods are more appropriate than the others.

**Figure 4:**
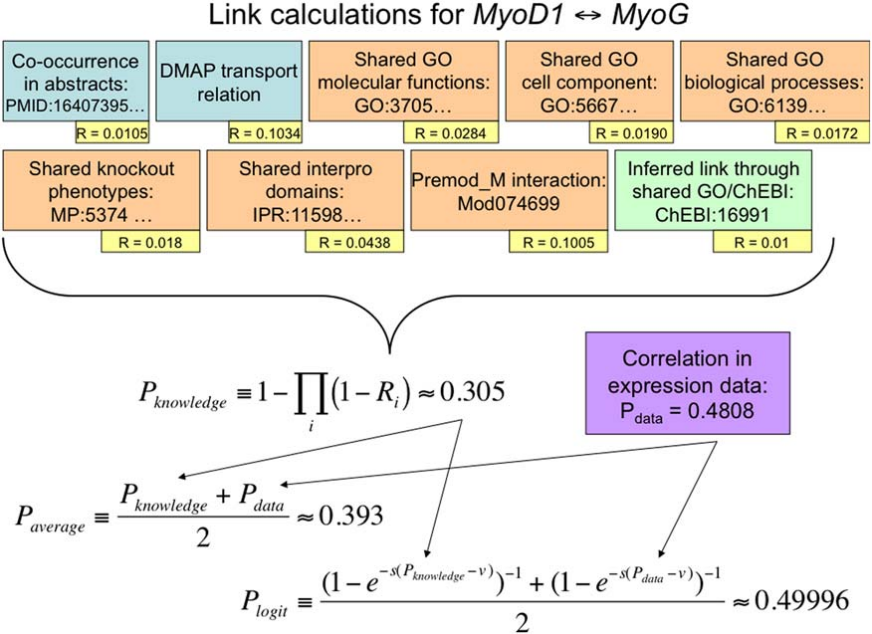
Creating a link in the combined network. This figure illustrates the creation of the link between *MyoD1* and *MyoG* in the combined network. Nine experts are illustrated, including two language processing experts (blue), six experts based on inference from shared ontology annotations or database entries (orange), and one based on shared components from enriched ontology annotations (green). Each expert has a computed reliability (yellow), computed as described in the text. The identifiers in the expert boxes indicate the provenance of the inferences, with ellipses indicating omissions for space. The correlation between the expression levels of these two genes in the experimental data, *P_data_*, is shown in purple. The Noisy-OR computation of the reliability from all knowledge sources is shown as *P_knowledge_* and the two functions that combine the knowledge and data networks are show as *P_average_* and *P_logit_*. In this case, *P_average_* was over the threshold for inclusion (top 1000 edges) in the combined grant, but *P_logit_* was not.

In the application described below, the distribution of weights in the knowledge and data graphs are such that the Averaging combination method gives high scores to arcs that are supported in both the data and knowledge networks (behaving somewhat like a Boolean AND), while the Logit method privileges the high scoring arcs in the data network over those in the knowledge network (since the distribution of correlations is weighted more towards 1 than the distribution of knowledge confidence scores). Figure 4 shows in detail how an example link is created in each of these graphs.

## Results

Use of the Hanalyzer is demonstrated in the analysis of an experiment that created a comprehensive expression dataset for mouse craniofacial development. The transcriptome of C57BL/6J strain (Jackson Labs) mice was sampled at 12 hour intervals from E10.5-E12.5, a time period that spans from formation of the facial prominences to when they fuse together to form the mature facial platform. Microdissected samples from three distinct facial regions were isolated at each time-point: the frontonasal, the maxillary, and the mandibular prominence. Seven independent biological replicates were prepared and analyzed for each sample. This dataset and an initial analysis of it are described in detail in [10]. To create the data network, the expression level of all the replicates at a particular time point and tissue for all probes associated with a particular MGI identifier are averaged. These averages are normalized by computing the log_2_ ratio of each gene's average expression level at each time point and tissue to the median expression level across all time points and tissues. The Pearson correlation coefficients over time and tissue are then computed for all pairs of genes.

**Figure 5:**
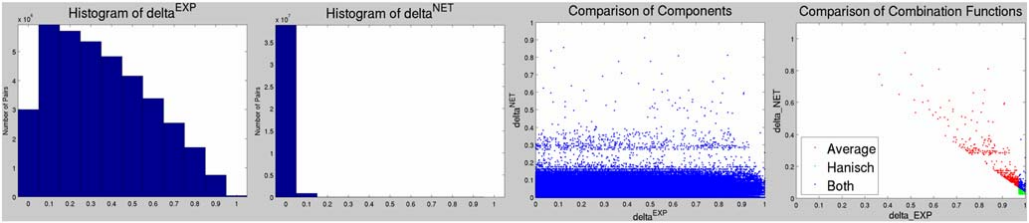
Comparison of network probabilities and their combinations. A) Histogram of edge probabilities for the experimental data component *P_EXP_* (y-axis is 106 scale). B) Histogram of edge probabilities for the network component P*_NET_* (y-axis is 107 scale). C) Scatterplot comparing the two probability distributions where each point represents an edge. D) Top 1000 scoring edges by either Average or Logit combination functions.

Two combined networks are created; one using edge Averaging and one using the Logit method. Arcs were included in a combined network only if at least three of the reading experts support it. Genes not linked to any other genes were removed, creating combined networks containing 8923 MGI identifiers. The arcs in these two combined networks were further pruned so that only the highest scoring 1000 edges by each method were visualized. Figure 5a–c illustrates the distributions of the individual components while Figure 5d illustrates the top 1000 edges for both Average and Logit combination networks.

As shown in Figure 5, the distributions of probabilities in the knowledge and data networks interact with the combination functions to achieve different sorts of reporting goals. The arcs that appear in the Average combination network are strongly connected in the background knowledge and in the data network. Identifying these already well-understood aspects of the data provides rapid orientation to an analyst. Using Cytoscape with our visualization plugin, an analyst can identify important functional themes rapidly, surveying details such as associated GO annotations, gene descriptions and known knock out phenotype information. In contrast, the edges that appear in the Logit combination network but not the Average network indicate links that are strong in the experimental data, but have only modest support in the background knowledge. These edges are used to generate new hypotheses about the roles of genes not previously known to be involved in the phenomena under study.

### Characterization of a representative sub-network

**Figure 6:**
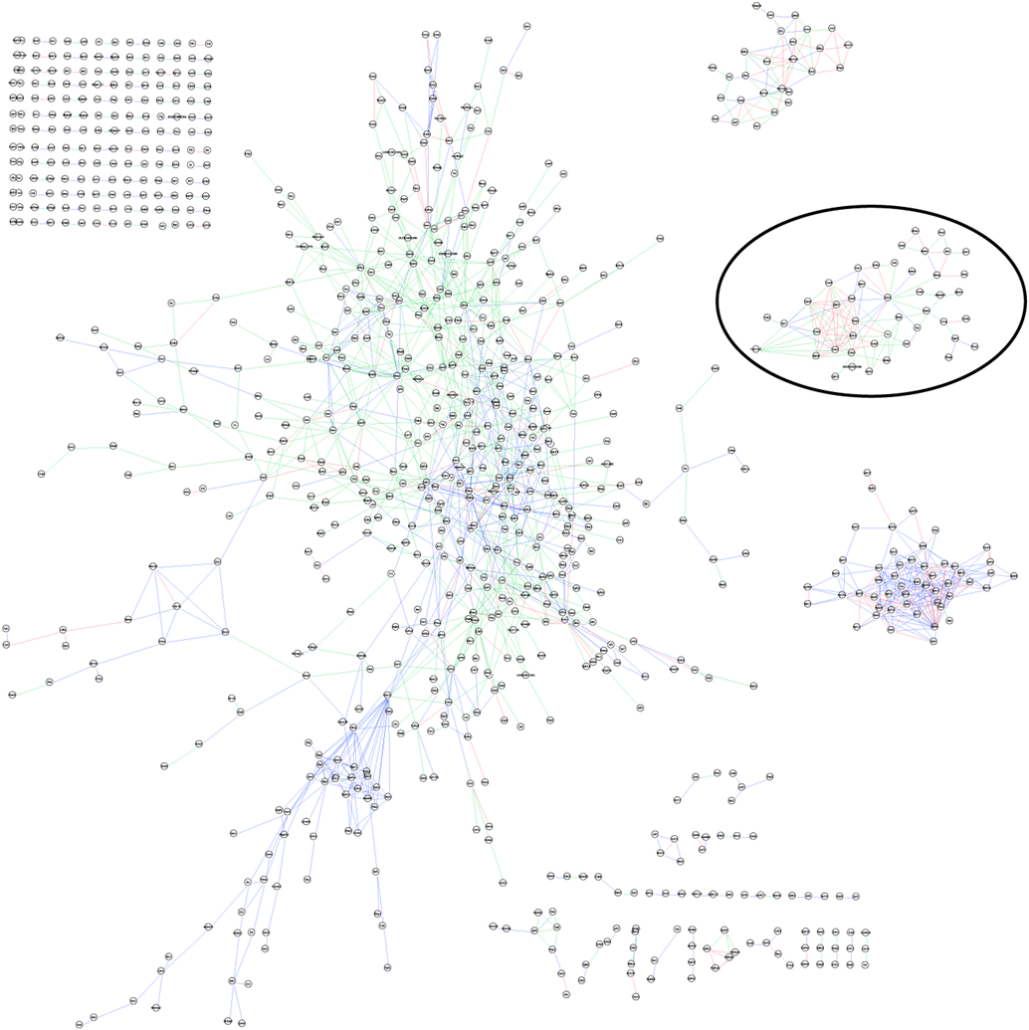
Graph of top 1000 edges asserted by the combination of expression data and background biological knowledge. Graph illustrating the network generated by taking the highest scoring 1000 edges as asserted by the Average (blue edges) and/or Logit (green edges) combinatorial measures. Blue edges indicate those edges asserted by both Average and Logit metrics. A total of 945 unique nodes (genes) and 1743 edges are shown (visualized in Cytoscape). The circled medium-sized sub-cluster to the right of the graph forms the basis of the investigations presented here (Figure 7).

The use of two different combination functions to investigate the network enables the development of an investigative methodology that supports hypothesis generation through systematic network exploration. The top 1000 edges as scored by either function generate a network comprised of 945 genes and 1,743 total edges. This collection of high scoring edges is organized as 92 pairs, 15 triplets, seven small clusters (<10 nodes), one large ‘yarnball’ (551 nodes), and three medium-sized clusters (comprising 27 to 51 nodes) (Figure 6). One of the medium-sized sub-networks (total 45 nodes, 107 edges, is analyzed in detail here (circled in Figure 6), illustrating a typical use of the Hanalyzer.

#### Sub-network explanation guided by the Average combination network

**Figure 7:**
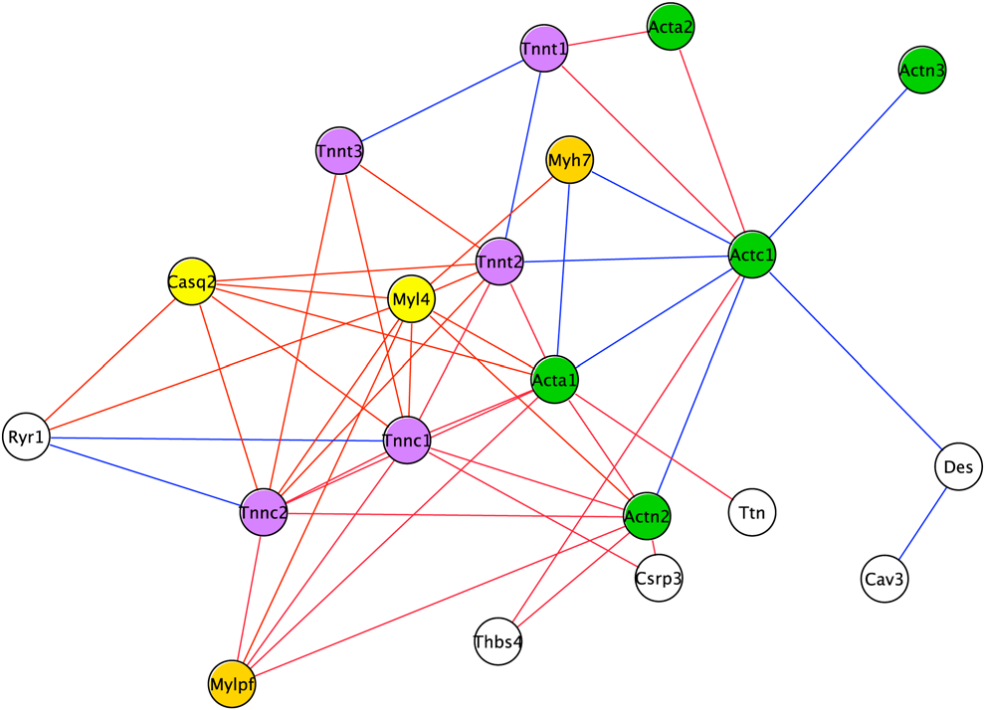
Sub-network comprising of edges asserted by the Average combinatorial metric. Graph illustrating the sub-network generated by viewing only those edges asserted by the Average combinatorial metric. A total of 20 nodes and 50 edges are present; blue edges indicate those asserted solely by the Average metric, while red edges indicate those asserted by both Average and Logit metrics. Nodes are labeled by gene symbol with different node colors representing different protein families (Myosin, yellow; Actin, green; Troponin, purple). Colorless nodes indicate no common protein family.

That sub-network contains 50 edges from the Average combination graph, involving 20 nodes (Figure 7); 15 edges asserted solely by the Average metric and 35 asserted by both the Average and Logit measures. By browsing the annotations associated with these 20 genes and their protein products it quickly became apparent that the theme common to this sub-network is muscle (Table S1). Nineteen of the 20 nodes have at least one reference to ‘muscle’ within their annotations or description, with the most informative descriptive terms being the GO Biological Process terms “muscle contraction” GO:0006936 (and children, including “regulation of muscle contraction” GO:0006937) and “muscle development” GO:0007517, together annotating 15 of the 20 nodes. It is also of interest to note that the majority of the nodes (13 of 20) in this network belong to one of three well characterized muscle protein families (Actin, Myosin and Troponin), suggesting that this network is involved in force generation and structural integrity of muscle.

The single apparent exception to this muscle theme was *Thbs4* (Thrombospondin 4, MGI:1101779). Direct searching of PubMed identified a role for Thbs4 (also known as TSP-4) in muscle formation. Thbs4 is secreted by developing tendon mesenchyme cells, and is part of a local signaling process involving the protein ankyrin repeat domain 1 (*Ankrd1*; MGI:1097717) which couples tendon morphogenesis to muscle formation [86] (note that *Ankrd1* was called “muscle ankyrin repeat protein” or *marp* in that paper). *Thbs4* is expressed at high levels (and in complementary patterns) to *Ankrd1* during myogenesis through late embryogenesis and is still observed postnatally [86].

**Figure 8:**
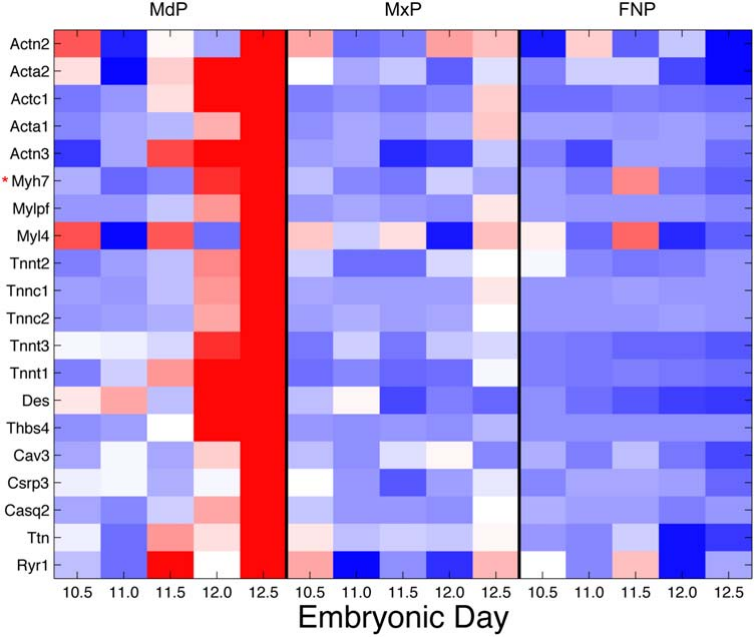
Heatmap of genes in the Average sub-network. Relative expression of each gene is shown across five time points and three tissues, with red indicating higher expression and blue lower. Genes are grouped by protein family and clustered within these functional groups. Genes whose expression was classed as ‘absent’ in >99% of the samples are indicated by a red * and are included here for completion.

This network is intriguing because of its strong muscle theme and because the expression profile of the nodes within this network is striking in its mandibular specificity (Figure 8). The expression of this group of 20 genes is consistently and exclusively up-regulated in the mandibular sample as development progresses from E10.5–12.5. The literature indicates that this expression profile is consistent with tongue muscle development; the tongue being the largest single muscle mass in the head and located within the mandible. At approximately E11, the migration of myogenic cells from the occipital somites into the tongue primordia is considered complete, with myoblasts continuing to proliferate and differentiate until around E15 when they fuse and withdraw from the cell cycle [87]. Desmin (*Des*, MGI:94885) mRNA is detected as early as E10, consistent with its marking early steps in skeletal myogenesis, such as myoblast determination [88]. Also, *Thbs4* has been shown to promote myogenic differentiation specifically in the tongue, which due to its lack of cartilage, links muscle groups through a tendinous scaffold [86].

This same group of genes is also up-regulated at the later E12–12.5 time point in the maxilla sample, consistent with a later onset of all other muscle cell differentiation in relation to the tongue. Skeletal muscle development is staggered, with the tongue maturing approximately 1.5 days (in mice) earlier than all other skeletal muscles. The more advanced stage of tongue muscle development at birth is thought to correlate with its requirement for mammalian suckling immediately after birth [88]. The lack of significant muscle in the frontonasal prominence accounts for the low level of expression of these genes in that tissue. The systematically reported and easily explored collection of relevant background knowledge made the interpretation of this complex set of evidence regarding the broad developmental function of a complex group of interacting genes much more straightforward than it would have been using any other approach with which we are familiar.

#### Hypothesis generation guided by the Logit combination network

**Figure 9:**
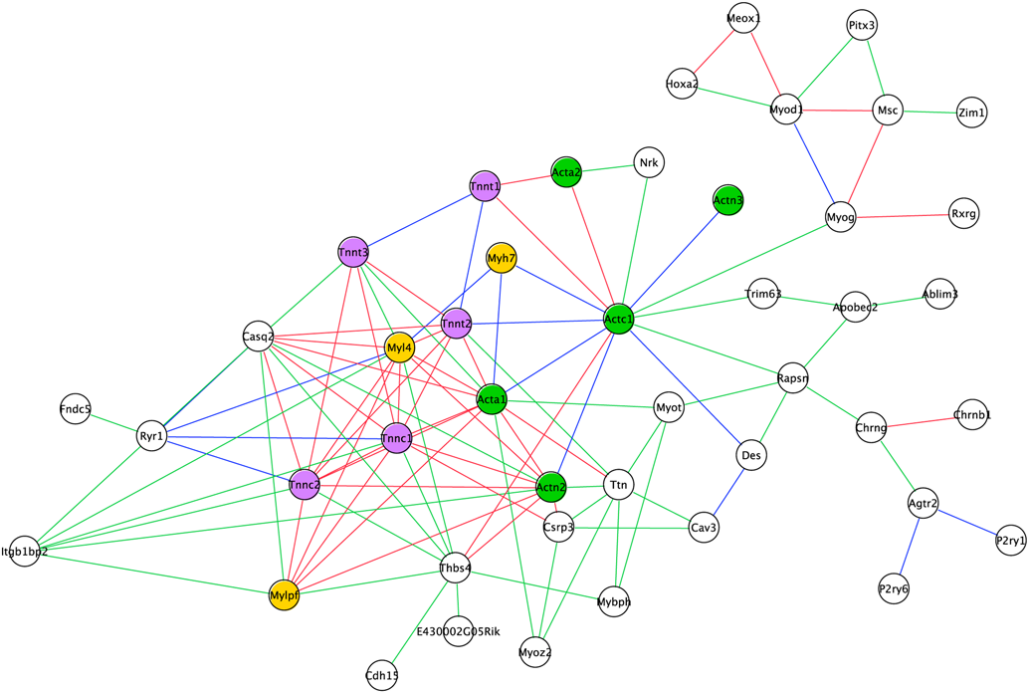
Sub-network comprising of edges asserted by both Average and Logit combined metrics. Graph illustrating the sub-network generated by viewing edges asserted by the both Average (blue edges) and Logit (green edges) combinatorial metrics. Red edges indicate those edges asserted by both metrics. Nodes are colored as previously described in Figure 7.

**Figure 10:**
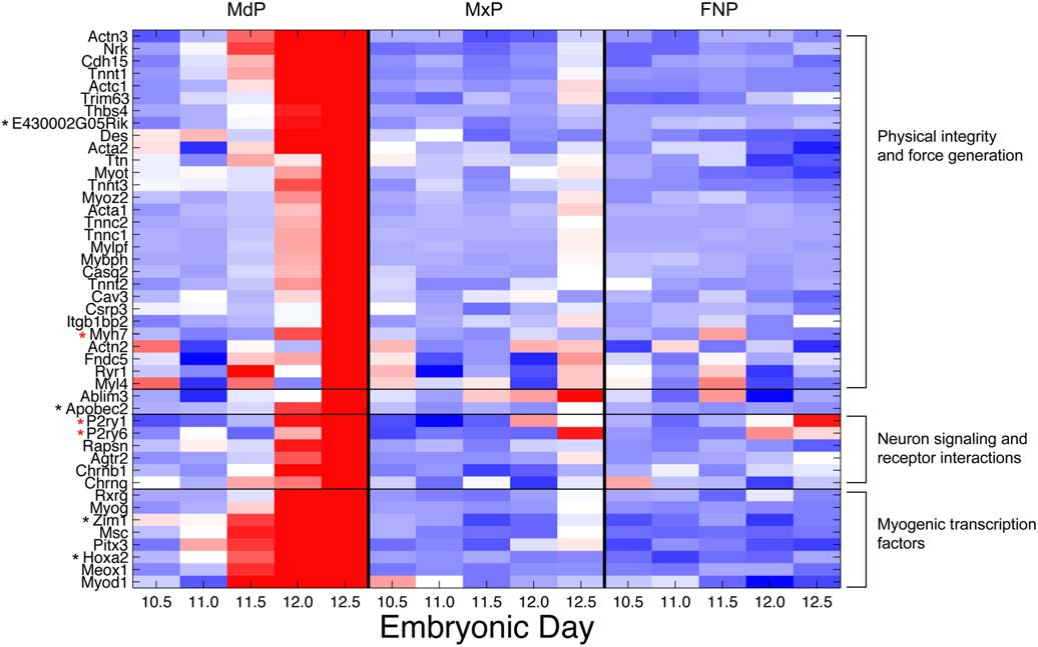
Heatmap of all genes in the sub-network. Relative expression of each gene is shown across five time points and three tissues, with red indicating higher expression and blue lower. Genes are grouped by function and clustered within these subgroups. Those genes highlighted as candidates are indicated by a black*. Those genes whose expression was classed as ‘absent’ in >99% of the samples are indicated by a red* and are included here for completion.

Once the well understood aspects of the sub-network had been explored and a biological explanation for the observations created, the analyst adds the edges asserted only by the Logit metric to the visualization of the sub-network. The inclusion of Logit-asserted edges introduced an additional 25 nodes to the network (total 45 nodes), and expanded the network to 107 edges (Figure 9). These 107 edges consist of 48 Logit-only edges, 18 Average edges (note the additional 3 Average edges linked into the network via connection to nodes introduced by the Logit edges) and 41 edges asserted by both Logit and Average metrics. The nodes comprising this larger network display the same striking mandible-specific expression pattern of the Average combination network, suggesting these additional nodes may also be implicated in tongue development (Figure 10).

**Figure 11:**
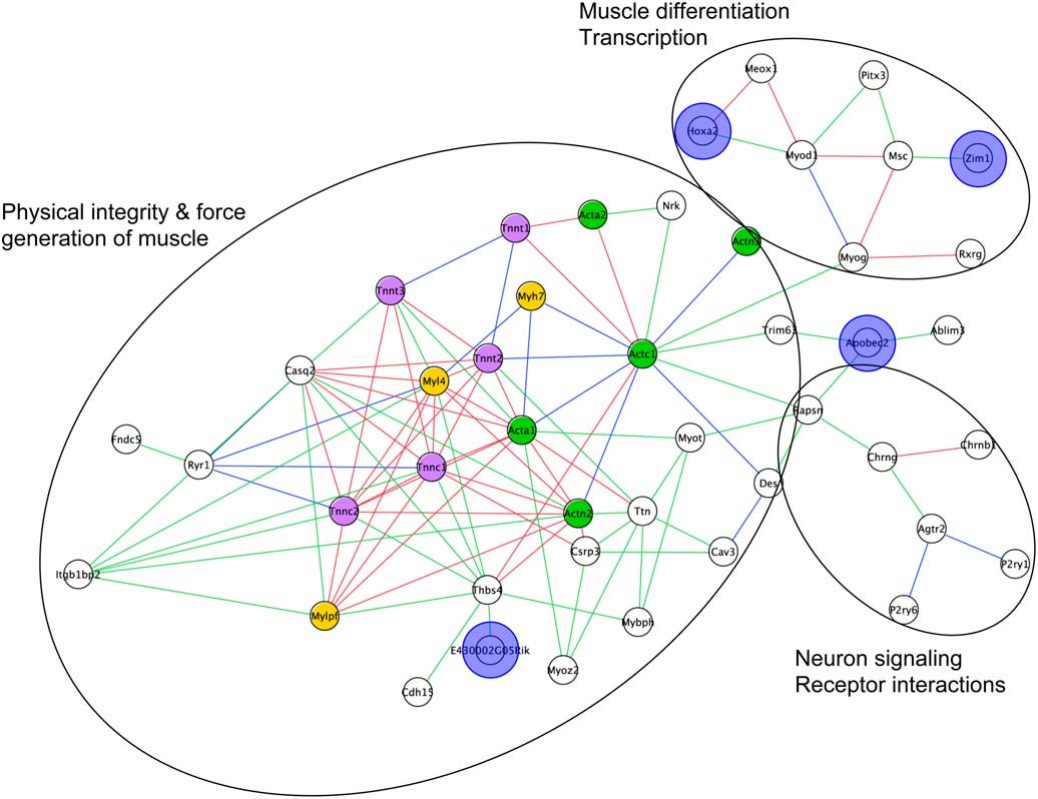
Functional clusters of nodes within the mandibular specific sub-network. The subnetwork can be seen to be separated into three functional clusters; physical integrity and generation of muscle, muscle differentiation and transcription, and neuron signaling and receptor interactions. Nodes and edges are colored as previously described in Figure 9. Nodes subject to further biological investigations are highlighted by opaque blue circles.

Although nine of these additional nodes expand the core cluster described above, the majority of nodes form two new clusters tethered to the initial group by one to four edges. Browsing the collated annotations associated with these additional nodes allowed rapid insight into common functional themes. These annotations indicated that the two additional clusters represent myogenic differentiation (six nodes) and synapse interactions (eight nodes) (Figure 11 and Table S2). Within the synapse cluster the most informative annotations are the KEGG annotation “Neuroactive ligand-receptor interaction” KEGG:mmu04080 and the GO Cellular Component term “postsynaptic membrane” GO:0045211, which together annotate all six members of this cluster. All eight nodes within the transcription cluster are, unsurprisingly, annotated with the GO Biological Process “transcription” GO:0006350, and five of these nodes also have a documented muscle-related knock out phenotype. The specific genes and interactions in each of these three clusters are explored in turn, and several are selected for experimental validation.

The first cluster investigated we called the *core cluster*. Of the nine additional nodes contributing to the structural cluster, four (*Cdh15*, *Nrk*, *Fndc5*, and *E430002G05Rik*; MGI:106672, MGI:1351326, MGI:1917614 and MGI:2445082, respectively) lack annotations from our experts suggesting a role in either muscle, or more generally, craniofacial development. Supplementary investigation of the literature and publicly available expression data was required to extrapolate the muscle association of these four genes.

In contrast to the other ‘unannotated’ nodes, *Cdh15* (also known as M-Cadherin, M denoting muscle [89]) is a very well studied gene with a number of associated publications (23 references tied to its MGI record alone [accessed 4/23/2008]). It has long been known that *Cdh15* is expressed in myogenic cells and has a role in skeletal muscle differentiation, as indicated by low level expression in skeletal myoblasts followed by an increased expression in myotube forming cells [89]. Its precise role during muscle development and regeneration is yet to be determined however, and a recent *Cdh15* null mouse model with apparently normal muscle phenotype suggesting functional compensation by other cadherin proteins [90].

The lack of information linking *Cdh15* with muscle development highlights the persisting problem of organism-specific gene name normalization. While *Cdh15* is the only official gene symbol, there are two approved names for the resultant protein product; Cadherin 15 and M-Cadherin (myotubule) [Data from HUGO, www.genenames.org Accessed 5/1/2008], and to confuse things further, both names are only used in the human records for this gene (Both GeneBank [NM_004933] and Entrez Gene [ID: 1013] use “Homo sapiens cadherin 15, M-cadherin (myotubule) (CDH15), mRNA” as their definition).

The literature indicates that the Ste20-type kinase, NIK-related kinase (*Nrk*) is predominantly expressed in developing skeletal musculature from E10.5 through E17 during mouse embryogenesis; however, *Nrk* expression is not detected in any adult tissues, including skeletal muscles [91]. Limited RNA expression data obtained from GenePaint.org [92], also appears to show *Nrk* expression in E14.5 tongue (GenePaint set ID: MH1818, section Embryo_C1818_1_4B).

In the developing embryo, the recently characterized fibronectin type III domain containing 5 gene (*Fndc5*, also known as *PeP* and *Pxp*; data from iHop [93]) is almost exclusively expressed in developing skeletal muscle [94]. Absent at E7, *Fndc5* expression is first detected in whole embryos at E11, and at E13.5 is specifically observed in the tongue and other skeletal muscles [94]. A role during myoblast differentiation is indicated by a two-fold increase in expression during the transition from myoblasts into myotubes, after which expression stabilizes and continues into and throughout adulthood [94].

Finally, investigation of the Riken clone *E430002G05Rik* presented little informative annotation. A single GeneRif identified from the associated EntrezGene entry (GeneID: 210622) yielded all information ascertained about this gene via the associated publication. This single publication [95] identified mRNAs affected in a mouse model (*mdx*) for Duchenne muscular dystrophy (DMD). *E430002G05Rik* was identified as a down-regulated transcript in the *mdx* mouse and subsequently named *RAMP* (Regeneration-associated muscle protease homolog) [95]. It was observed that *RAMP* is predominantly expressed in normal adult skeletal muscle and brain, and that it is specifically up-regulated in regenerating skeletal muscle fibers after injury [95]. The absence of any annotation regarding development prompted the selection of this gene for further experimental validation.

We called the second cluster explored the *Transcription Factor Cluster*. Although well annotated as transcription factors, information provided by reading experts on *Pitx3*, *Rxrg* and *Zim1* (MGI:1100498, MGI:98216, and MGI:1341879, respectively) did not suggest roles in muscle development (Table S2), prompting further investigations. *Pitx3* is well characterized and annotated with respect to its role in lens formation during eye development [96],[97]. However, literature searching revealed that tongue-specific expression of *Pitx3* (also known as *Ptx3*) during development (expression first detected at E11.5) was documented over a decade ago [98], while its specific role in myogenesis and myoblast differentiation has only more recently been reported [99].

Known and annotated principally for its role in mediating the effects of retinoic acid, there also exists extensive literature associating *Rxrg* (retinoid X receptor gamma) with myoblast differentiation. This association was not asserted by any of the reading experts, although 117 papers were returned by PubMed search with query “rxr muscle” (accessed 4/25/2008), also suggesting difficulties in species-specific gene name normalization. As early as 1993, RXRs were identified as positive regulators of skeletal muscle development via their direct interactions with Myogenin and MyoD promotor elements [100],[101], and the role of *Rxrg* in muscle continues to be explored, with the most recent associated publication identifying a role in lipogenesis and SREBP1c regulation in skeletal muscle [102]. A high-throughput study identifying transcription units involved in brain development [103] indirectly documented the tongue-specific expression profile of *Rxrg* in E13.5 mice (image MGI:3507450), with the same expression pattern weakly persevering in E14.5 mice (GenePaint.org set ID: C1279, section Embryo_C1279_6_3D).

Significantly less is known about the zinc-finger gene, *Zim1*. In mouse, this gene is part of an imprinted cluster that includes *Zim2* (MGI:1923887) and *Peg3* (MGI:104748) [104], but a *Zim1* ortholog has not been identified to date in human. Therefore, it has been proposed that *Zim1* is a recent addition to the mouse genome that was derived via a local duplication of *Zim2*. In mice, *Zim1* is maternally imprinted and is only expressed during embryogenesis, notably in the limb bud and therefore it has been suggested as having a role in limb development [105]. Limited and unannotated RNA expression information was available from additional studies in the mouse [103]; however, these did not address *Zim1* expression in the developing face. We therefore selected *Zim1* for experimental validation, as there was only limited knowledge of this gene and its function in mouse facial and muscle development.

Although well studied in craniofacial development, we also selected *Hoxa2* (MGI:96174) for further analysis as its expression is not normally associated with branchial arch 1, which gives rise to the mandible. Indeed, *Hoxa2* has a strong anterior limit of expression in the neural crest cells originating in rhombomere 4 that generate the mesenchyme of the second branchial arch. Moreover, the absence of Hox gene expression in more rostral tissues, including the first branchial arch, has been postulated to have enabled the evolution of the vertebrate head [106],[107],[108],[109],[110],[111],[112]. We therefore decided to explore this potential novel domain of *Hoxa2* expression in more detail.

The third cluster explored was called the *synapse cluster*. All the nodes contributing to the synapse cluster are unambiguously implicated in neuromuscular signaling. However, two additional nodes (*Ablim3* and *Apobec2*; MGI:2442582 and MGI:1343178 respectively) fail to fit neatly into any cluster, and instead appear to straddle the synapse interaction and muscle structure clusters. *Ablim3* annotation includes both the GO Molecular Function term “actin binding” GO:0003779 as well as the KEGG annotation “Axon guidance” KEGG:mmu04360. However, the annotation associated with *Apobec2* strongly indicates a role in RNA editing and processing, but gives no indication of a role in muscle (Table S2).

The *Apobec2*-associated literature revealed little consensus regarding its function. *Apobec2* has been documented as an ancestral, cardiac and skeletal muscle-specific member of the *Apobec* family implicated in muscle regeneration [113]. It has also been described as a ubiquitously expressed protein with cytidine deaminase RNA editing activity [114]. Apobec2 knockout mice appear viable and fertile [113] but no examination of the tongue was reported. *Apobec2* was selected for further biological investigation due to the sparse nature of current associated knowledge and its possible function in the tongue muscle development.

### Experimental testing of the generated hypotheses

The above analysis generated hypotheses regarding the role of four genes (*Apobec2*, *E430002G05Rik*, *Hoxa2*, *Zim1*) in the development of the murine tongue. These hypotheses were tested by whole-mount in situ hybridizations to E11.5 and E12.5 mouse embryos, collected, prepared and hybridized as described in [115], stained with *Hoxa2* [116], *Apobec2*, *E430002G05Rik* and *Zim3* RNA probes, as described in [117]. The mouse *Apobec2* probe was derived by PCR from E10.5 FVB mouse head cDNA using the primers Apobec2F (5′-CCA GCC AGG CTT AGC TGC TGA CAG-3′) and Apobec2R (5′-GCT CAC CAG AAT GAG CAG ACG AAG-3′); the mouse *E430002G05Rik* probe was derived using the primers E43F (5′-GGT TTA TCA TCC AGT TGA GGT TTG G-3′) and E43R (5′-GCA GAC AGG TTG CTT TCC TGA-3′); the mouse *Zim3* probe was derived using the primers Zim3F (5′-CGT ACA AGT GTG ACA AGT GC)-3′ and Zim3R (5′-GCA CAA ATG CTC CAA GTA GG-3′).

**Figure 12:**
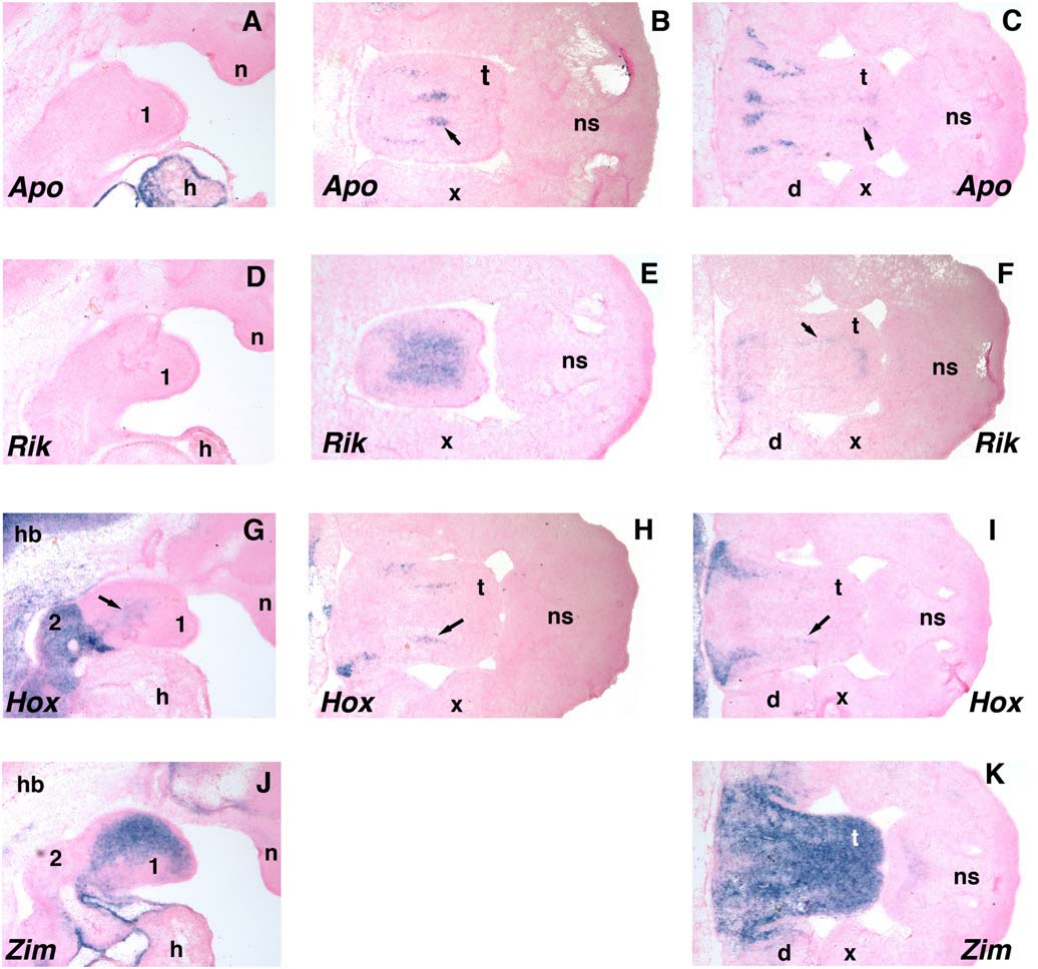
Gene expression in the developing mouse tongue. *In situ* hybridization using anti-sense probes for *Apobec2* (A–C), *E430002G05Rik* (D–F), *Hoxa2* (G–I), and *Zim1* (J, K). (A, D, G, J) sagittal sections of an E11.5 head; (B, C, E, F, H, I) are transverse sections of an E12.5 head. Anterior is to the right on all panels. Dark staining represents hybridization signal from the probe, the pink color is from a histological counterstain. The arrows indicate areas of fainter staining. (B, E, H) are more rostral sections than (C, F and I). The tongue has a mild convexity at these stages of development, being raised on its rostral aspect (see panels J). Therefore, more rostral sections will tend to skim the midsection of the tongue at the surface. More caudal sections will tend to intersect with staining patterns at their anterior and posterior domains (compare panels B and C). *Apobec2* and *E430002G05Rik* did not generate significant tongue staining at E11.5. Control experiments using sense probes did not yield specific staining. 1, mandibular component of first branchial arch (future lower jaw as well as future anterior and middle of tongue); 2, second branchial arch (future posterior, lateral, part of tongue – major site of *Hoxa2* expression); d, mandible; h, heart; hb, hindbrain; n, nasal prominence; ns, nasal septum; t, tongue; x, maxillary process.

As shown in Figure 12 all four genes are expressed in the developing tongue at E12.5. Examination of the first arch tissue at E11.5 indicates that neither *Apobec2* nor *E430002G05Rik* are expressed at this time-point, although the former gene is clearly expressed in the developing cardiac region (A, D). However, by E12.5 both genes are expressed in discrete regions of the developing tongue (B,C, E, F). At E11.5 *Hoxa2* expression is prominent in the second arch tissue and there is clearly a sharp boundary of expression with the first arch (G). Nevertheless, weaker expression is apparent in the core of the first arch, and expression is again visible in the tongue at E12.5, presenting as bilateral stripes aligned with the anterioposterior axis (H, I). *Zim1* expression is visible in the core mesenchyme of the first branchial arch at E11.5 and by E12.5 almost the entire tongue, with the exception of the ectoderm, is strongly stained.

This data confirms all four hypotheses; each of these genes is expressed in the developing tongue. The expression patterns for the four genes are different though, indicating that their function may not be directly related. The almost uniform expression of *Zim1* at E12.5 suggests that it is marking the neural crest derived mesenchyme of the tongue that will give rise to the smooth muscle and connective tissue. Alternatively, it may identify the intrinsic skeletal musculature of the tongue. In contrast, the expression of the other three genes is consistent with their expression in different extrinsic tongue muscles that project out of the tongue and attach to surrounding skeletal components to allow tongue movement during swallowing and chewing.

## Discussion

The data we have obtained for the four genes we analyzed in detail, *Apobec2*, *E430002G05Rik*, *Hoxa2*, and *Zim1*, indicate that all four were indeed expressed in the developing mandible, specifically in the tongue. Further analysis will be required to determine if these genes have specific roles in tongue development and function, and if they act as specific markers for individual components of the intrinsic and extrinsic tongue musculature. Nevertheless, two observations are worth noting with respect to the expression patterns of *Zim1* and *Hoxa2*. First, the expression of *Zim1* in the tongue has considerable overlap with that of the linked *Peg3* gene [118] and the expression profiles of these two genes are also very similar in the microarray dataset we have obtained. Unfortunately, data were not available on the linked *Zim2* gene in our analysis [10] because the single probe set in the array did not generate a reliable signal. We hypothesize that the presence of *Zim1* in our network is due to the importance of the linked *Peg3* gene, and that the expression of *Zim1* reflects its recent insertion next to the cis-regulatory sequences responsible for *Peg3* expression.

It is worth noting that *Peg3* was in the data network, but the scores of the arcs linking it to *Zim1* were below the top 1000 threshold used in both the Average and Logit combined networks. Expanding the reporting component to provide an option to visualize all linkages passing a threshold correlation in the data network alone might have proven useful here. This example also provides a caution for analysts: genes can appear in the combination networks for many reasons, not all of which indicate a causal role in the phenomena under study.

With respect to *Hoxa2*, we do detect expression of this gene in the first branchial arch, although the level is considerably less than documented for the second arch. Whether this expression pattern marks cells intrinsic to the first arch migrating in from the second arch remains to be determined. We also note that *Hoxa2* mutant mice have a number of craniofacial defects associated with the developing tongue. Specifically, in *Hoxa2*-null mice the tongue is not able to move appropriately during embryonic development and its abnormal location prevents closure of the secondary palate. Available data indicates that this is due to the absence of neural crest derived skeletal elements originating from the second branchial arch which function as the attachment sites for extrinsic tongue muscles [119],[120]. Our findings that *Hoxa2* is also expressed in these extrinsic tongue muscles raises the possibility that the loss of *Hoxa2* may directly cause tongue muscle defects leading to cleft palate.

Explaining the biological phenomena underlying complex, high-throughput datasets in light of existing knowledge is a critical step in the exploitation of powerful post-genomic instrumentation, as is generation of new, biologically significant hypotheses. This application of the Hanalyzer demonstrates that 3R systems have the potential to facilitate these analyses, making apparently overwhelming amounts of background knowledge particularly useful for analysts, accelerating the pace of biomedical discovery.

Inference to the best explanation (sometimes called abduction) is a complex task that can involve many other forms of reasoning. Although related to determination of causes, explanations can involve non-causal factors as well, and not all causal factors may be important in a particular explanation [121]. One particularly important sort of explanation in biomedicine is the contrastive explanation (why this *rather than* that), which is well suited to the carefully controlled experimental methodology that underlies biomedical research. The system described here does not automate the production of explanations (nor hypotheses), but provides a novel class of software support for human users who are doing so.

### Current limitations and future work

The version of the Hanalyzer described here built a background network for only mouse genes, and the data network was constructed from a particularly well-powered time and tissue gene expression array series. One important question is how well this methodology will generalize to other organisms and data types. [36] demonstrated many of the experts that compose this system can be used to build knowledge networks for other model organisms, including yeast, worm, and fly. Ongoing work involves building knowledge networks for human and rat as well. As many of the same types of experts are available for each of these organisms, expansion of the knowledge networks to other organisms is a straightforward software engineering task.

One important issue in generalizing to other organisms is the question of how to build homology-based experts. As observed in [122],[123],[124], there are many factors that are involved in the successful application of homology-based protein-protein interaction networks. Such predictions are even more difficult and uncertain in humans and other eukaryotes, although promising methods that could form the basis for such experts have been published recently, e.g. [7] and [125].

The natural language processing aspects of the system increase its performance over database integration systems alone [32]. The OpenDMAP approach used is state of the art [126], but there is much room for improvement. One particularly important area for future work is in multi-organism gene normalization.

While there is a great deal of data in the form of expression arrays over which gene correlations can be made, there are many other sources of high-throughput information that could be profitably analyzed using 3R methodology. Construction of quantitative data networks with genes or gene products as fiducials could be based on data produced by many high throughput experimental techniques, including proteomics, miRNA assays, genotyping, and others. What are the best methods for generating such data networks, and are there differences in the types of knowledge networks (experts) that are best suited to analyzing them?

Another issue regards the inherently changing nature of biomedical knowledge. Experts can be re-run periodically to keep the knowledge networks up to date, but a variety of open research questions about handling time remain: Is it valuable to highlight more recent results for annotators? How should temporal considerations factor into the reliability calculations? Should reasoning experts take temporal considerations into account? How?

While the experts used in the Hanalyzer proved to be useful for analysts, there are a large number of potential experts, both reading (external) and reasoning that could be included in a 3R system. What is the optimal set of experts to use for building knowledge networks? Does that differ for different applications? The Noisy-OR combination method (and most others) assumes that the experts are independent of each other, yet many potentially useful sources of knowledge exhibit complex dependencies; should selection of experts be made in light of this constraint? Many sorts of inference, ranging from logical entailment to information theoretic, statistical or heuristic might be productively included in a 3R system; what is the optimal set of reasoning experts to use?

Finally, while the example of biological validation of several hypotheses generated through the use of the system provides some evidence that the system is of genuine value to biological data analysts, the question of how best to evaluate 3R systems remains open. Perhaps the “insight-based” evaluation methodology previously described for scientific visualization systems [127],[128] could be modified to evaluate 3R systems as well.

### Availability

The Hanalyzer, including the experts and the Cytoscape plugin for visualization is available as open source software via SourceForge at hanalyzer.sourceforge.net. The extracted assertions from the OpenDMAP text mining experts are available as supplementary materials associated with [4]; the links from the ACF expert are available as supplementary materials associated with [32].

## Supporting Information

Table S1 Annotation terms associated with nodes within the Average network.
(0.08 MB DOC)

Table S2 Annotation terms associated with those nodes added via the Logit asserted edges.
(0.09 MB DOC)

## References

[1] Baumgartner WA, Cohen KB, Fox LM, Acquaah-Mensah G, Hunter L (2007). Manual curation is not sufficient for annotation of genomic databases.. Bioinformatics.

[2] Hunter L, Cohen K (2006). Biomedical language processing: what's beyond PubMed?. Molecular Cell.

[3] Galperin MY (2008). The Molecular Biology Database Collection: 2008 update.. Nucleic Acids Res.

[4] Hunter L, Lu Z, Firby J, Baumgartner WA, Johnson HL (2008). OpenDMAP: an open source, ontology-driven concept analysis engine, with applications to capturing knowledge regarding protein transport, protein interactions and cell-type-specific gene expression.. BMC Bioinformatics.

[5] Eisenberg D, Marcotte EM, Xenarios I, Yeates TO (2000). Protein function in the post-genomic era.. Nature.

[6] Salwinski L, Miller CS, Smith AJ, Pettit FK, Bowie JU (2004). The Database of Interacting Proteins: 2004 update.. Nucleic Acids Res.

[7] Scott MS, Barton GJ (2007). Probabilistic prediction and ranking of human protein-protein interactions.. BMC Bioinformatics.

[8] Karimpour-Fard A (2008).

[9] Leach S (2006).

[10] Feng W, Leach S, Tipney H, Phang T, Geraci M (2008). Spatial and Temporal Analysis of Gene Expression During Growth and Fusion of the Mouse Facial Prominences.. In Preparation.

[11] Bellazzi R, Zupan B (2007). Towards knowledge-based gene expression data mining.. J Biomed Inform.

[12] Subramanian A, Tamayo P, Mootha VK, Mukherjee S, Ebert BL (2005). Gene set enrichment analysis: a knowledge-based approach for interpreting genome-wide expression profiles.. Proc Natl Acad Sci U S A.

[13] Daigle BJ, Altman RB (2008). M-BISON: microarray-based integration of data sources using networks.. BMC Bioinformatics.

[14] Reiss DJ, Avila-Campillo I, Thorsson V, Schwikowski B, Galitski T (2005). Tools enabling the elucidation of molecular pathways active in human disease: application to Hepatitis C virus infection.. BMC Bioinformatics.

[15] Sivachenko AY, Yuryev A, Daraselia N, Mazo I (2007). Molecular networks in microarray analysis.. J Bioinform Comput Biol.

[16] Daraselia N, Yuryev A, Egorov S, Novichkova S, Nikitin A (2004). Extracting human protein interactions from MEDLINE using a full-sentence parser.. Bioinformatics.

[17] Ideker T, Ozier O, Schwikowski B, Siegel AF (2002). Discovering regulatory and signalling circuits in molecular interaction networks.. Bioinformatics.

[18] Eppig JT, Blake JA, Bult CJ, Kadin JA, Richardson JE (2007). The mouse genome database (MGD): new features facilitating a model system.. Nucleic Acids Res.

[19] Bader GD, Donaldson I, Wolting C, Ouellette BF, Pawson T (2001). BIND–The Biomolecular Interaction Network Database.. Nucleic Acids Res.

[20] Xenarios I, Salwinski L, Duan XJ, Higney P, Kim SM (2002). DIP, the Database of Interacting Proteins: a research tool for studying cellular networks of protein interactions.. Nucleic Acids Res.

[21] Zanzoni A, Montecchi-Palazzi L, Quondam M, Ausiello G, Helmer-Citterich M (2002). MINT: a Molecular INTeraction database.. FEBS Lett.

[22] Kerrien S, Alam-Faruque Y, Aranda B, Bancarz I, Bridge A (2007). IntAct–open source resource for molecular interaction data.. Nucleic Acids Res.

[23] Suzuki H, Fukunishi Y, Kagawa I, Saito R, Oda H (2001). Protein-protein interaction panel using mouse full-length cDNAs.. Genome Res.

[24] Drabkin HJ, Hollenbeck C, Hill DP, Blake JA (2005). Ontological visualization of protein-protein interactions.. BMC Bioinformatics.

[25] Ashburner M, Ball CA, Blake JA, Botstein D, Butler H (2000). Gene ontology: tool for the unification of biology. The Gene Ontology Consortium.. Nat Genet.

[26] Wingender E, Dietze P, Karas H, Knuppel R (1996). TRANSFAC: a database on transcription factors and their DNA binding sites.. Nucleic Acids Res.

[27] Ferretti V, Poitras C, Bergeron D, Coulombe B, Robert F (2007). PReMod: a database of genome-wide mammalian cis-regulatory module predictions.. Nucleic Acids Res.

[28] Schlitt T, Palin K, Rung J, Dietmann S, Lappe M (2003). From Gene Networks to Gene Function.. Genome Research.

[29] Alako BT, Veldhoven A, van Baal S, Jelier R, Verhoeven S (2005). CoPub Mapper: mining MEDLINE based on search term co-publication.. BMC Bioinformatics.

[30] Bowers PM, Pellegrini M, Thompson MJ, Fierro J, Yeates TO (2004). Prolinks: a database of protein functional linkages derived from coevolution.. Genome Biol.

[31] Ramani AK, Bunescu RC, Mooney RJ, Marcotte EM (2005). Consolidating the set of known human protein-protein interactions in preparation for large-scale mapping of the human interactome.. Genome Biol.

[32] Gabow A, Leach S, Baumgartner WA, Hunter L, Goldberg D (2008). Improving protein function prediction methods with integrated literature data.. BMC Bioinformatics.

[33] Krallinger M, Morgan A, Smith L, Leitner F, Tanabe L (2008). Evaluation of text-mining systems for biology: overview of the Second BioCreative community challenge.. Genome Biology.

[34] Kanehisa M, Goto S, Kawashima S, Okuno Y, Hattori M (2004). The KEGG resource for deciphering the genome.. Nucleic Acids Res.

[35] Lord PW, Stevens RD, Brass A, Goble CA (2003). Semantic similarity measures as tools for exploring the gene ontology.. Pac Symp Biocomput.

[36] Leach S, Gabow A, Hunter L, Goldberg DS (2007). Assessing and combining reliability of protein interaction sources.. Pac Symp Biocomput.

[37] Bada M, Hunter L (2007). Enrichment of OBO ontologies.. J Biomed Inform.

[38] Hill DP, Blake JA, Richardson JE, Ringwald M (2002). Extension and integration of the gene ontology (GO): combining GO vocabularies with external vocabularies.. Genome Res.

[39] Chen Y, Xu D (2003). Computational analyses of high-throughput protein-protein interaction data.. Curr Protein Pept Sci.

[40] Marcotte EM, Pellegrini M, Thompson MJ, Yeates TO, Eisenberg D (1999). A combined algorithm for genome-wide prediction of protein function.. Nature.

[41] Saito R, Suzuki H, Hayashizaki Y (2003). Construction of reliable protein-protein interaction networks with a new interaction generality measure.. Bioinformatics.

[42] Goldberg DS, Roth FP (2003). Assessing experimentally derived interactions in a small world.. Proc Natl Acad Sci U S A.

[43] Chen J, Hsu W, Lee ML, Ng SK (2006). Increasing confidence of protein interactomes using network topological metrics.. Bioinformatics.

[44] Pei P, Zhang A (2005). A topological measurement for weighted protein interaction network.. Proc IEEE Comput Syst Bioinform Conf.

[45] Jansen R, Yu H, Greenbaum D, Kluger Y, Krogan NJ (2003). A Bayesian networks approach for predicting protein-protein interactions from genomic data.. Science.

[46] Troyanskaya OG, Dolinski K, Owen AB, Altman RB, Botstein D (2003). A Bayesian framework for combining heterogeneous data sources for gene function prediction (in Saccharomyces cerevisiae).. Proc Natl Acad Sci U S A.

[47] Myers CL, Robson D, Wible A, Hibbs MA, Chiriac C (2005). Discovery of biological networks from diverse functional genomic data.. Genome Biol.

[48] Segal E, Wang H, Koller D (2003). Discovering molecular pathways from protein interaction and gene expression data.. Bioinformatics.

[49] Imoto S, Higuchi T, Goto T, Tashiro K, Kuhara S (2003). Combining microarrays and biological knowledge for estimating gene networks via Bayesian networks.. Proc IEEE Comput Soc Bioinform Conf.

[50] Jaimovich A, Elidan G, Margalit H, Friedman N (2006). Towards an integrated protein-protein interaction network: a relational Markov network approach.. J Comput Biol.

[51] Nariai N, Kim S, Imoto S, Miyano S (2004). Using protein-protein interactions for refining gene networks estimated from microarray data by Bayesian networks.. Pac Symp Biocomput.

[52] Cui J, Li P, Li G, Xu F, Zhao C (2008). AtPID: Arabidopsis thaliana protein interactome database–an integrative platform for plant systems biology.. Nucleic Acids Res.

[53] Li J, Li X, Su H, Chen H, Galbraith DW (2006). A framework of integrating gene relations from heterogeneous data sources: an experiment on Arabidopsis thaliana.. Bioinformatics.

[54] Yeang CH, Ideker T, Jaakkola T (2004). Physical network models.. J Comput Biol.

[55] Myers CL, Troyanskaya OG (2007). Context-sensitive data integration and prediction of biological networks.. Bioinformatics.

[56] Lanckriet GR, De Bie T, Cristianini N, Jordan MI, Noble WS (2004). A statistical framework for genomic data fusion.. Bioinformatics.

[57] Vert JP, Kanehisa M (2003). Extracting active pathways from gene expression data.. Bioinformatics.

[58] Yamanishi Y, Vert JP, Kanehisa M (2004). Protein network inference from multiple genomic data: a supervised approach.. Bioinformatics.

[59] Deane CM, Salwinski L, Xenarios I, Eisenberg D (2002). Protein interactions: two methods for assessment of the reliability of high throughput observations.. Mol Cell Proteomics.

[60] Edwards AM, Kus B, Jansen R, Greenbaum D, Greenblatt J (2002). Bridging structural biology and genomics: assessing protein interaction data with known complexes.. Trends Genet.

[61] Asthana S, King OD, Gibbons FD, Roth FP (2004). Predicting protein complex membership using probabilistic network reliability.. Genome Res.

[62] Bader JS, Chaudhuri A, Rothberg JM, Chant J (2004). Gaining confidence in high-throughput protein interaction networks.. Nat Biotechnol.

[63] Sprinzak E, Sattath S, Margalit H (2003). How reliable are experimental protein-protein interaction data?. J Mol Biol.

[64] Lee I, Date SV, Adai AT, Marcotte EM (2004). A probabilistic functional network of yeast genes.. Science.

[65] Nabieva E, Jim K, Agarwal A, Chazelle B, Singh M (2005). Whole-proteome prediction of protein function via graph-theoretic analysis of interaction maps.. Bioinformatics.

[66] von Mering C, Jensen LJ, Snel B, Hooper SD, Krupp M (2005). STRING: known and predicted protein-protein associations, integrated and transferred across organisms.. Nucleic Acids Res.

[67] Sun J, Sun Y, Ding G, Liu Q, Wang C (2007). InPrePPI: an integrated evaluation method based on genomic context for predicting protein-protein interactions in prokaryotic genomes.. BMC Bioinformatics.

[68] Hishigaki H, Nakai K, Ono T, Tanigami A, Takagi T (2001). Assessment of prediction accuracy of protein function from protein–protein interaction data.. Yeast.

[69] Hwang D, Rust AG, Ramsey S, Smith JJ, Leslie DM (2005). A data integration methodology for systems biology.. Proc Natl Acad Sci U S A.

[70] Cozman F (2004). Axiomatizing Noisy-OR, Technical Report from Escola Politecnica da USP.. BT/PMR/0409.

[71] Chuang HY, Lee E, Liu YT, Lee D, Ideker T (2007). Network-based classification of breast cancer metastasis.. Mol Syst Biol.

[72] Faloutsos C, McCurley K, Tomkins A

[73] Dupont P, Jerome C, Dooms G, Monette J, Deville Y (2006). Relevant subgraph extratcion from random walks in a graph.. Research Report RR.

[74] Bader GD, Hogue CW (2003). An automated method for finding molecular complexes in large protein interaction networks.. BMC Bioinformatics.

[75] Shannon P, Markiel A, Ozier O, Baliga N, Wang J (2003). Cytoscape: a software environment for integrated models of biomolecular interaction networks.. Genome Research.

[76] Tipney H, Leach S, Feng W, Spritz R, Williams T (2008). Leveraging existing biological knowledge in the identification of candidate genes for facial dysmorphology..

[77] Draghici S, Khatri P, Tarca AL, Amin K, Done A (2007). A systems biology approach for pathway level analysis.. Genome Res.

[78] Sohler F, Hanisch D, Zimmer R (2004). New methods for joint analysis of biological networks and expression data.. Bioinformatics.

[79] Yamanishi Y, Vert JP, Nakaya A, Kanehisa M (2003). Extraction of correlated gene clusters from multiple genomic data by generalized kernel canonical correlation analysis.. Bioinformatics.

[80] Hanisch D, Zien A, Zimmer R, Lengaur T (2002). Co-clustering of biological networks and gene expresion data.. Bioinformatics.

[81] Nakaya A, Goto S, Kanehisa M (2001). Extraction of correlated gene clusters by multiple graph comparison.. Genome Inform.

[82] Ogata H, Fujibuchi W, Goto S, Kanehisa M (2000). A heuristic graph comparison algorithm and its application to detect functionally related enzyme clusters.. Nucleic Acids Res.

[83] Iossifov I, Krauthammer M, Friedman C, Hatzivassiloglou V, Bader JS (2004). Probabilistic inference of molecular networks from noisy data sources.. Bioinformatics.

[84] Rhodes DR, Tomlins SA, Varambally S, Mahavisno V, Barrette T (2005). Probabilistic model of the human protein-protein interaction network.. Nat Biotechnol.

[85] Hedges L, Olkin I (1985). Statistical Methods for Meta-Analysis.

[86] Baumeister A, Arber S, Caroni P (1997). Accumulation of muscle ankyrin repeat protein transcript reveals local activation of primary myotube endcompartments during muscle morphogenesis.. J Cell Biol.

[87] Yamane A, Mayo M, Shuler C, Crowe D, Ohnuki Y (2000). Expression of myogenic regulatory factors during the development of mouse tongue striated muscle.. Archives of Oral Biology.

[88] Amano A, Yamane A, Shimada M, Koshimizu U, Nakamura T (2002). Hepatocyte growth factor is essential for the migration of myogenic cells and promotes their proliferation during the early periods of tongue morphogenesis in mouse embryos.. Developmental Dynamics.

[89] Donalies M, Cramer M, Ringwald M, Starzinski-Powitz A (1991). Expression of M-cadherin, a member of the cadherin multigene family, correlates with differentiation of skeletal muscle cells.. Proc Natl Acad Sci U S A.

[90] Hollnagel A, Grund C, Franke WW, Arnold HH (2002). The cell adhesion molecule M-cadherin is not essential for muscle development and regeneration.. Mol Cell Biol.

[91] Kanai-Azuma M, Kanai Y, Okamoto M, Hayashi Y, Yonekawa H (1999). Nrk: a murine X-linked NIK (Nck-interacting kinase)-related kinase gene expressed in skeletal muscle.. Mech Dev.

[92] Visel A, Thaller C, Eichele G (2004). GenePaint.org: an atlas of gene expression patterns in the mouse embryo.. Nucleic Acids Res.

[93] Hoffman R, Valencia A (2004). A Gene Network for Navigating the Literature.. Nature Genetics.

[94] Ferrer-Martinez A, Ruiz-Lozano P, Chien KR (2002). Mouse PeP: a novel peroxisomal protein linked to myoblast differentiation and development.. Dev Dyn.

[95] Nakayama Y, Nara N, Kawakita Y, Takeshima Y, Arakawa M (2004). Cloning of cDNA encoding a regeneration-associated muscle protease whose expression is attenuated in cell lines derived from Duchenne muscular dystrophy patients.. Am J Pathol.

[96] Cvekl A, Tamm ER (2004). Anterior eye development and ocular mesenchyme: new insights from mouse models and human diseases.. Bioessays.

[97] Graw J (2003). The genetic and molecular basis of congenital eye defects.. Nat Rev Genet.

[98] Smidt MP, van Schaick HS, Lanctot C, Tremblay JJ, Cox JJ (1997). A homeodomain gene Ptx3 has highly restricted brain expression in mesencephalic dopaminergic neurons.. Proc Natl Acad Sci U S A.

[99] L'Honore A, Coulon V, Marcil A, Lebel M, Lafrance-Vanasse J (2007). Sequential expression and redundancy of Pitx2 and Pitx3 genes during muscle development.. Dev Biol.

[100] Downes M, Griggs R, Atkins A, Olson EN, Muscat GE (1993). Identification of a thyroid hormone response element in the mouse myogenin gene: characterization of the thyroid hormone and retinoid X receptor heterodimeric binding site.. Cell Growth Differ.

[101] Downes M, Mynett-Johnson L, Muscat GE (1994). The retinoic acid and retinoid X receptors are differentially expressed during myoblast differentiation.. Endocrinology.

[102] Kamei Y, Miura S, Suganami T, Akaike F, Kanai S (2008). Regulation of SREBP1c Gene Expression in Skeletal Muscle: Role of Retinoid X Receptor/Liver X Receptor and Forkhead-O1 Transcription Factor.. Endocrinology.

[103] Gray PA, Fu H, Luo P, Zhao Q, Yu J (2004). Mouse brain organization revealed through direct genome-scale TF expression analysis.. Science.

[104] Kim J, Bergmann A, Lucas S, Stone R, Stubbs L (2004). Lineage-specific imprinting and evolution of the zinc-finger gene ZIM2.. Genomics.

[105] Kim J, Lu X, Stubbs L (1999). Zim1, a maternally expressed mouse Kruppel-type zinc-finger gene located in proximal chromosome 7.. Hum Mol Genet.

[106] Gavalas A, Trainor P, Ariza-McNaughton L, Krumlauf R (2001). Synergy between Hoxa1 and Hoxb1: the relationship between arch patterning and the generation of cranial neural crest.. Development.

[107] Kanzler B, Kuschert SJ, Liu YH, Mallo M (1998). Hoxa-2 restricts the chondrogenic domain and inhibits bone formation during development of the branchial area.. Development.

[108] Yang X, Zhou Y, Barcarse EA, O'Gorman S (2008). Altered neuronal lineages in the facial ganglia of Hoxa2 mutant mice.. Dev Biol.

[109] Hunt P, Gulisano M, Cook M, Sham MH, Faiella A (1991). A distinct Hox code for the branchial region of the vertebrate head.. Nature.

[110] Rijli FM, Mark M, Lakkaraju S, Dierich A, Dolle P (1993). A homeotic transformation is generated in the rostral branchial region of the head by disruption of Hoxa-2, which acts as a selector gene.. Cell.

[111] Tan DP, Ferrante J, Nazarali A, Shao X, Kozak CA (1992). Murine Hox-1.11 homeobox gene structure and expression.. Proc Natl Acad Sci U S A.

[112] Gendron-Maguire M, Mallo M, Zhang M, Gridley T (1993). Hoxa-2 mutant mice exhibit homeotic transformation of skeletal elements derived from cranial neural crest.. Cell.

[113] Mikl MC, Watt IN, Lu M, Reik W, Davies SL (2005). Mice deficient in APOBEC2 and APOBEC3.. Mol Cell Biol.

[114] Matsumoto T, Marusawa H, Endo Y, Ueda Y, Matsumoto Y (2006). Expression of APOBEC2 is transcriptionally regulated by NF-kappaB in human hepatocytes.. FEBS Lett.

[115] Brewer S, Feng W, Huang J, Sullivan S, Williams T (2004). Wnt1-Cre-mediated deletion of AP-2 causes multiple neural crest-related defects.. Developmental Biology.

[116] Maconochie M, Krishnamurthy R, Nonchev S, Meier P, Manzanares M (1999). Regulation of Hoxa2 in cranial neural crest cells involves members of the AP-2 family.. Development.

[117] Feng W, Williams T (2003). Cloning and characterization of the mouse AP-2 epsilon gene: a novel family member expressed in the developing olfactory bulb.. Mol Cell Neurosci.

[118] Li L, Keverne EB, Aparicio SA, Ishino F, Barton SC (1999). Regulation of maternal behavior and offspring growth by paternally expressed Peg3.. Science.

[119] Barrow JR, Capecchi MR (1999). Compensatory defects associated with mutations in Hoxa1 restore normal palatogenesis to Hoxa2 mutants.. Development.

[120] Nazarali A, Puthucode R, Leung V, Wolf L, Hao Z (2000). Temporal and spatial expression of Hoxa-2 during murine palatogenesis.. Cell Mol Neurobiol.

[121] Lipton P (2004). Inference to the best explanation.

[122] Karimpour-Fard A, Detweiler CS, Erickson KD, Hunter L, Gill RT (2007). Cross-species cluster co-conservation: a new method for generating protein interaction networks.. Genome Biol.

[123] Karimpour-Fard A, Hunter L, Gill RT (2007). Investigation of factors affecting prediction of protein-protein interaction networks by phylogenetic profiling.. BMC Genomics.

[124] Karimpour-Fard A, Leach SM, Hunter LE, Gill RT (2008). The topology of the bacterial co-conserved protein network and its implications for predicting protein function.. BMC Genomics.

[125] Barker D, Meade A, Pagel M (2007). Constrained models of evolution lead to improved prediction of functional linkage from correlated gain and loss of genes.. Bioinformatics.

[126] Baumgartner WA, Lu Z, Johnson HL, Caporaso JG, Paquette J (2008). Concept recognition for extracting protein interaction relations from biomedical text.. Genome Biology.

[127] North C (2006). Toward measuring visualization insight.. IEEE Comput Graph Appl.

[128] Saraiya P, North C, Lam V, Duca KA (2006). An insight-based longitudinal study of visual analytics.. IEEE Trans Vis Comput Graph.

