# Supplementary material for: Biomedical Discovery Acceleration, with Applications to Craniofacial Development
Source: PLoS Comput Biol. 2009 Mar 27;5(3):e1000215. doi: 10.1371/journal.pcbi.1000215 (PMC2653649; doi:10.1371/journal.pcbi.1000215)
Supplement: Table S1 — Annotation terms associated with nodes within the Average network. (0.08 MB DOC) [file pcbi.1000215.s001.doc]

| Gene | Description | GO_BP | GO_CC | Phenotype |
| --- | --- | --- | --- | --- |
| Acta1 | actin, alpha 1, skeletal muscle | /muscle_contraction /muscle_development /muscle_thin_filament_assembly /skeletal_muscle_development /skeletal_muscle_fiber_development | /striated_muscle_thin_filament/ actin_filament/ actin_cytoskeleton | MP:0002106_abnormal muscle physiology |
| Acta2 | actin, alpha 2, smooth muscle, aorta |  |  |  |
| Actc1 | actin, alpha, cardiac | /muscle_contraction /muscle_development /regulation_of_heart_contraction | /actin_filament /actin_cytoskeleton | /MP:0000267_abnormal cardiac development/ MP:0000274_enlarged heart/ MP:0000278_abnormal myocardial fiber morphology/ MP:0000749_muscle degeneration/ MP:0002190_disorganized myocardium/ MP:0002625_left ventricle hypertrophy/ MP:0002754_dilated right ventricle/ MP:0003222_increased cardiomyocyte apoptosis/ MP:0005140_decreased cardiac muscle contractility/ MP:0005598_decreased ventricle muscle contractility |
| Actn2 | actinin alpha 2 | /muscle_contraction | /striated_muscle_thin_filament /Z_disc |  |
| Actn3 | actinin alpha 3 | /muscle_contraction | /striated_muscle_thin_filament |  |
| Casq2 | calsequestrin 2 | /regulation_of_muscle_contraction /striated_muscle_contraction |  |  |
| Cav3 | caveolin 3 |  |  | /MP:0000278_abnormal myocardial fiber morphology/ MP:0000281_abnormal ventricular septum morphology/ MP:0000749_muscle degeneration/ MP:0000751_myopathy/ MP:0000752_dystrophic muscle/ MP:0001625_cardiac hypertrophy/ MP:0001853_heart inflammation/ MP:0002625_left ventricle hypertrophy/ MP:0002753_dilated left ventricle/ MP:0002833_increased heart weight/ MP:0002953_thick ventricular wall/ MP:0003084_abnormal skeletal muscle fiber morphology/ MP:0003141_cardiac fibrosis/ MP:0003852_skeletal muscle necrosis/ MP:0004121_abnormal sarcolemma morphology/ MP:0004150_absent caveolae/ MP:0005330_cardiomyopathy/ MP:0005598_decreased ventricle muscle contractility/ MP:0005608_cardiac interstitial fibrosis |
| Csrp3 | cysteine and glycine-rich protein 3 | /striated_muscle_development | /Z_disc | /MP:0000274_enlarged heart/ MP:0001625_cardiac hypertrophy/ MP:0002753_dilated left ventricle/ MP:0002795_dilated cardiomyopathy/ MP:0005140_decreased cardiac muscle contractility/ MP:0005598_decreased ventricle muscle contractility/ MP:0005599_increased cardiac muscle contractility/ MP:0005608_cardiac interstitial fibrosis |
| Des | desmin | /muscle_development | /myofibril /Z_disc /sarcolemma /contractile_fiber | MP:0000278_abnormal myocardial fiber morphology/ MP:0000749_muscle degeneration/ MP:0000759_abnormal skeletal muscle morphology/ MP:0000761_thin diaphragm muscle/ MP:0000767_abnormal smooth muscle morphology/ MP:0001625_cardiac hypertrophy/ MP:0002190_disorganized myocardium/ MP:0002279_abnormal diaphragm morphology/ MP:0002332_abnormal exercise endurance/ MP:0002652_thin myocardial wall/MP:0002753_dilated left ventricle/ MP:0002754_dilated right ventricle/ MP:0002837_dystrophic cardiac calcinosis/ MP:0003141_cardiac fibrosis/ MP:0004084_abnormal cardiac muscle relaxation/ MP:0005329_abnormal cardiac muscle morphology/ MP:0005330_cardiomyopathy/ MP:0005592_abnormal vascular smooth muscle morphology/ MP:0005598_decreased ventricle muscle contractility/ MP:0006085_myocardial necrosis/ |
| Myh7 | myosin, heavy polypeptide 7, cardiac muscle, beta | /striated_muscle_contraction /muscle_development | /striated_muscle_thick_filament /myosin |  |
| Myl4 | myosin, light polypeptide 4 |  | /myosin |  |
| Mylpf | myosin light chain, phosphorylatable, fast skeletal muscle |  | /myosin |  |
| Ryr1 | ryanodine receptor 1, skeletal muscle | /regulation_of_muscle_contraction |  | MP:0000759_abnormal skeletal muscle morphology/ MP:0002106_abnormal muscle physiology/ MP:0002114_abnormal axial skeleton morphology/ MP:0002841_impaired skeletal muscle contractility/ MP:0003084_abnormal skeletal muscle fiber morphology/ MP:0005620_abnormal muscle contractility/ |
| Thbs4 | thrombospondin 4 |  |  |  |
| Tnnc1 | troponin C, cardiac/slow skeletal | /regulation_of_muscle_contraction |  |  |
| Tnnc2 | troponin C2, fast | /regulation_of_muscle_contraction |  |  |
| Tnnt1 | troponin T1, skeletal, slow | /regulation_of_muscle_contraction |  |  |
| Tnnt2 | troponin T2, cardiac | /muscle_contraction | /sarcomere |  |
| Tnnt3 | troponin T3, skeletal, fast | /regulation_of_muscle_contraction |  |  |
| Ttn | titin | sarcomere_alignment /muscle_development | /muscle_myosin /sarcomere /Z_disc /M_line | MP:0000278_abnormal myocardial fiber morphology/ MP:0000748_progressive muscle weakness/ MP:0000749_muscle degeneration/ MP:0001865_striated muscle inflammation/ MP:0002652_thin myocardial wall/ MP:0004090_abnormal sarcomere morphology/ MP:0004094_abnormal M lines/ MP:0005140_decreased cardiac muscle contractility/ MP:0005329_abnormal cardiac muscle morphology/ MP:0005369_muscle phenotype/ |

**Table S1: Annotation terms associated with nodes within the Average network.**

The table highlights annotation evidence of nodes within the Average network which supports a muscle theme. GO Biological Process (GO_BP) and Cellular Component (GO_CC) annotation terms and associated phenotypes (extracted from MGI) are shown here and, due to the high number of annotations, only those indicating a muscle association are listed. As can be seen, all 19 of the 20 nodes have at least one indication of a relationship with muscle; Thbs4 being the exception.
