# Supplementary material for: Biomedical Discovery Acceleration, with Applications to Craniofacial Development
Source: PLoS Comput Biol. 2009 Mar 27;5(3):e1000215. doi: 10.1371/journal.pcbi.1000215 (PMC2653649; doi:10.1371/journal.pcbi.1000215)
Supplement: Table S2 — Annotation terms associated with those nodes added via the Logit asserted edges. (0.09 MB DOC) [file pcbi.1000215.s002.doc]

|  | **Gene** | **Description** | **GO_BP** | **GO_MF** | **GO_CC** | **Phenotype** | **KEGG** | **InterPro** |
| --- | --- | --- | --- | --- | --- | --- | --- | --- |
| **Muscle structural integrity and contractile force** | Cdh15 | cadherin 15 | /cell_adhesion /homophilic_cell_adhesion | /calcium_ion_binding /protein_binding | /extracellular_space /membrane /integral_to_membrane |  | /Cell adhesion molecules (CAMs) | /IPR000233_Cadherin cytoplasmic region /IPR002126_Cadherin |
|  | E430002G05Rik* | RIKEN cDNA E430002G05 gene | /proteolysis | /chymotrypsin_activity/trypsin_activity /peptidase_activity | /extracellular_space |  |  | /IPR000436_Sushi or SCR or CCP /IPR000742_EGF-like, type 3/IPR000859_CUB /IPR001254_Peptidase S1 and S6, chymotrypsin or Hap /IPR001314_Peptidase S1A, chymotrypsin /IPR006209_EGF-like/IPR006210_EGF /IPR009003_Peptidase, trypsin-like serine and cysteine /IPR013032_EGF-like region |
|  | Fndc5 | fibronectin type III domain containing 5 | /protein_targeting | /protein_binding | /integral_to_membrane |  | /ECM-receptor interaction | /IPR003961_Fibronectin, type III /IPR008957_Fibronectin, type III-like fold |
|  | Itgb1bp2 | integrin beta 1 binding protein 2 |  | /calcium_ion_binding /zinc_ion_binding | **/Z_disc** | /MP:0001544_abnormal cardiovascular system physiology |  | /IPR007051_CHORD /IPR007052_CS/IPR008978_HSP20-like chaperone |
|  | Mybph | **myosin binding** protein H | **/striated_muscle_contraction**  /cell_adhesion | /protein_binding | **/striated_muscle_thick_filament** |  |  | /IPR003598_Immunoglobulin subtype 2/IPR003599_Immunoglobulin subtype/IPR003961_Fibronectin, type III/IPR003962_Fibronectin, type III subdomain/IPR007110_Immunoglobulin-like/IPR008957_Fibronectin, type III-like fold/IPR013098_Immunoglobulin I-set |
|  | Myot | myotilin |  | **/actin_binding** /structural_molecule_activity /protein_binding | /cytoskeleton |  |  | /IPR003598_Immunoglobulin subtype 2/IPR003599_Immunoglobulin subtype/IPR007110_Immunoglobulin-like/IPR013098_Immunoglobulin I-set |
|  | Myoz2 | myozenin 2 |  | /structural_molecule_activity | **/actin_cytoskeleton /Z_disc** | /MP:0000274_enlarged heart  **/MP:0000278_abnormal myocardial fiber morphology /MP:0000759_abnormal skeletal muscle morphology** /MP:0001625_cardiac hypertrophy /MP:0002753_dilated left ventricle /MP:0002754_dilated right ventricle **/MP:0002972_abnormal cardiac muscle contractility** /MP:0003140_dilated atria /MP:0005330_cardiomyopathy |  | /IPR008438_Calcineurin-binding |
|  | Nrk | Nik related kinase | /protein_amino_acid_phosphorylation /activation_of_JNKK_activity | /nucleotide_binding /protein_kinase_activity /protein_serine or threonine_kinase_activity /protein-tyrosine_kinase_activity /small_GTPase_regulator_activity /ATP_binding /kinase_activity /transferase_activity /transferase_activity,_transferring_phosphorus-containing_groups /phosphotransferase_activity,_alcohol_group_as_acceptor |  |  |  | /IPR000719_Protein kinase /IPR001180_Citron-like /IPR002290_Serine or threonine protein kinase /IPR008271_Serine or threonine protein kinase, active site /IPR011009_Protein kinase-like |
|  | Trim63 | tripartite motif-containing 63 | /ubiquitin_cycle **/muscle_contraction** /proteasomal_ubiquitin-dependent_protein_catabolism | /ubiquitin-protein_ligase_activity /protein_binding /zinc_ion_binding /ligase_activity /small_conjugating_protein_ligase_activity /metal_ion_binding | /intracellular /nucleus **/myofibril /contractile_fiber** | **/MP:0002269_muscular atrophy** |  | /IPR000315_Zinc finger, B-box /IPR001841_Zinc finger, RING-type /IPR003649_B-box, C-terminal |
|  | Ablim3 | **actin binding** LIM protein family, member 3 | /cytoskeleton_organization_and_biogenesis | **/actin_binding** /zinc_ion_binding /metal_ion_binding |  |  | **/Axon guidance** | /IPR001781_LIM, zinc-binding /IPR003128_Villin headpiece |
|  | Apobec2* | apolipoprotein B editing complex 2 | /mRNA_editing /mRNA_processing | /RNA_editase_activity /zinc_ion_binding /hydrolase_activity /hydrolase_activity,_acting_on_carbon-nitrogen_but_not_peptide_bonds,_in_cyclic_amidines /metal_ion_binding |  | /MP:0000063_reduced bone density /MP:0001262_decreased body weight /MP:0001732_postnatal growth retardation /MP:0002896_abnormal bone mineralization | /Atrazine degradation | /IPR002125_CMP or dCMP deaminase, zinc-binding /IPR007904_APOBEC-like, C-terminal /IPR013158_APOBEC-like, N-terminal |
| **Neuron and synapse associations** | Agtr2 | angiotensin II receptor, type 2 | /angiotensin_mediated_vasodilation_during_regulation_of_blood_pressure /brain_renin-angiotensin_system /cell_communication /signal_transduction /G-protein_coupled_receptor_protein_signaling_pathway | /rhodopsin-like_receptor_activity /purinergic_nucleotide_receptor_activity /signal_transducer_activity /receptor_activity /transmembrane_receptor_activity /G-protein_coupled_receptor_activity /angiotensin_type_II_receptor_activity /bradykinin_receptor_activity /purinergic_nucleotide_receptor_activity,_G-protein_coupled | /membrane /integral_to_membrane | /MP:0000230_abnormal blood pressure /MP:0000343_altered response to myocardial infarction /MP:0001402_hypoactivity /MP:0001417_decreased exploration in new environment /MP:0001424_abnormal water consumption /MP:0001489_decreased startle reflex /MP:0001622_abnormal vasculogenesis /MP:0001657_abnormal induced morbidity or mortality /MP:0002753_dilated left ventricle /MP:0002842_increased blood pressure /MP:0002975_vascular smooth muscle hypertrophy /MP:0003043_hypoalgesia /MP:0003459_increased fear-related response /MP:0005534_decreased body temperature /MP:0005588_increased vasoconstriction **/MP:0005592_abnormal vascular smooth muscle morphology /MP:0005593_abnormal vascular smooth muscle contraction** /MP:0006143_increased diastolic blood pressure /MP:0006144_increased systolic blood pressure | **/Neuroactive ligand-receptor interaction** | /IPR000248_Angiotensin II receptor /IPR000276_Rhodopsin-like GPCR superfamily /IPR001186_B1 bradykinin receptor /IPR002453_Beta tubulin |
|  | Chrnb1 | cholinergic receptor, nicotinic, beta polypeptide 1 **(muscle)** | /transport /ion_transport | /signal_transducer_activity/receptor_activity /transmembrane_receptor_activity /nicotinic_acetylcholine-activated_cation-selective_channel_activity /ion_channel_activity /extracellular_ligand-gated_ion_channel_activity /channel_or_pore_class_transporter_activity /ligand-gated_ion_channel_activity **/neurotransmitter_receptor_activity** | /extracellular_space /Golgi_apparatus /plasma_membrane /membrane /integral_to_membrane **/postsynaptic_membrane** |  |  | /IPR002394_Nicotinic acetylcholine receptor **/IPR006029_Neurotransmitter-gated ion-channel transmembrane region /IPR006201_Neurotransmitter-gated ion-channel /IPR006202_Neurotransmitter-gated ion-channel ligand-binding** |
|  | Chrng | cholinergic receptor, nicotinic, gamma polypeptide | /transport /ion_transport **/synaptic_transmission** | /signal_transducer_activity /receptor_activity /transmembrane_receptor_activity /nicotinic_acetylcholine-activated_cation-selective_channel_activity /ion_channel_activity /extracellular_ligand-gated_ion_channel_activity /channel_or_pore_class_transporter_activity /ligand-gated_ion_channel_activity **/neurotransmitter_receptor_activity** | /Golgi_apparatus /plasma_membrane /membrane /integral_to_membrane **/postsynaptic_membrane** | /MP:0000755_hindlimb paralysis **/MP:0001052_abnormal muscle innervation /MP:0001053_abnormal neuromuscular synapse /MP:0001078_abnormal phrenic nerve** /MP:0001429_dehydration **/MP:0001436_abnormal suckling behavior** /MP:0001575_cyanosis **/MP:0001951_abnormal breathing** /MP:0002081_perinatal lethality **/MP:0002106_abnormal muscle physiology** /MP:0002279_abnormal diaphragm morphology **/MP:0002914_abnormal endplate potential activity /MP:0003084_abnormal skeletal muscle fiber morphology /MP:0005369_muscle phenotype** /MP:0005386_behavior or neurological phenotype |  | /IPR002394_Nicotinic acetylcholine receptor **/IPR006029_Neurotransmitter-gated ion-channel transmembrane region /IPR006201_Neurotransmitter-gated ion-channel /IPR006202_Neurotransmitter-gated ion-channel ligand-binding** |
|  | P2ry1 | purinergic receptor P2Y, G-protein coupled 1 | /cell_communication /signal_transduction /G-protein_coupled_receptor_protein_signaling_pathway | /rhodopsin-like_receptor_activity /purinergic_nucleotide_receptor_activity /signal_transducer_activity/receptor_activity /transmembrane_receptor_activity /G-protein_coupled_receptor_activity /C-X-C_chemokine_receptor_activity purinergic_nucleotide_receptor_activity,_G-protein_coupled | /integral_to_plasma_membrane /membrane /integral_to_membrane | /MP:0002551_abnormal blood coagulation /MP:0003422_abnormal thrombolysis /MP:0005464_abnormal platelet physiology /MP:0005606_increased bleeding time | **/Neuroactive ligand-receptor interaction** | /IPR000276_Rhodopsin-like GPCR superfamily /IPR001277_C-X-C chemokine receptor, type 4 /IPR002286_P2 purinoceptor |
|  | P2ry6 | pyrimidinergic receptor P2Y, G-protein coupled, 6 | /cell_communication /signal_transduction /G-protein_coupled_receptor_protein_signaling_pathway /transepithelial_chloride_transport | /rhodopsin-like_receptor_activity /purinergic_nucleotide_receptor_activity /signal_transducer_activity/receptor_activity /transmembrane_receptor_activity /G-protein_coupled_receptor_activity /pyrimidine_nucleotide_binding /purinergic_nucleotide_receptor_activity,_G-protein_coupled /UDP-activated_nucleotide_receptor_activity | /membrane /integral_to_membrane |  | **/Neuroactive ligand-receptor interaction** | /IPR000276_Rhodopsin-like GPCR superfamily /IPR002286_P2 purinoceptor |
|  | Rapsn | receptor-associated protein **of the synapse** |  | /signal_transducer_activity /receptor_activity /transmembrane_receptor_activity /binding /protein_binding /zinc_ion_binding /metal_ion_binding | /Golgi_apparatus /cytoskeleton /plasma_membrane **/synapse /postsynaptic_membrane** | **/MP:0000747_muscle weakness /MP:0001053_abnormal neuromuscular synapse /MP:0001055_failure of postsynaptic differentiation** /MP:0001954_respiratory distress /MP:0002081_perinatal lethality |  | /IPR001440_Tetratricopeptide TPR_1 /IPR001841_Zinc finger, RING-type /IPR008940_Protein prenyltransferase /IPR011595_Tetratricopeptide-related region /IPR011990_Tetratricopeptide-like helical /IPR013026_Tetratricopeptide region |
| **Transcription and differentiation** | Hoxa2* | homeo box A2 | **/cell_fate_determination /transcription /transcription,_DNA-dependent /regulation_of_transcription,_DNA-dependent /development** /pattern_specification /regulation_of_cellular_metabolism /middle_ear_morphogenesis **/regulation_of_transcription /embryonic_viscerocranium_morphogenesis** | /DNA_binding **/transcription_factor_activity** /sequence-specific_DNA_binding | /nucleus **/transcription_factor_complex** | **/MP:0000079_abnormal basioccipital bone morphology /MP:0000111_cleft palate /MP:0000438_abnormal skull morphology** /MP:0000841_abnormal hindbrain morphology **/MP:0000966_reduced sensory neuron number /MP:0001436_abnormal suckling behavior** /MP:0001953_respiratory failure /MP:0002058_neonatal lethality /MP:0002177_abnormal outer ear morphology **/MP:0004136_abnormal tongue muscle morphology** /MP:0005105_abnormal middle ear ossicle morphology /MP:0005579_absent outer ear |  | /IPR000047_Helix-turn-helix motif, lambda-like repressor /IPR001356_Homeobox /IPR001827_Homeobox protein, antennapedia type /IPR009057_Homeodomain-like /IPR012287_Homeodomain-related |
|  | Meox1 | mesenchyme homeobox 1 | /somite_specification **/transcription /transcription,_DNA-dependent /regulation_of_transcription,_DNA-dependent** /development /regulation_of_cellular_metabolism **/regulation_of_transcription** | /DNA_binding **/transcription_factor_activity** /sequence-specific_DNA_binding | /nucleus **/transcription_factor_complex** | /MP:0000138_absent vertebrae /MP:0000151_absent rib **/MP:0000733_abnormal muscle development** /MP:0001258_decreased body length /MP:0001688_abnormal somite development /MP:0002058_neonatal lethality /MP:0002080_embryonic lethality /MP:0002632_vestigial tail /MP:0002823_abnormal rib development /MP:0005225_abnormal vertebrae development |  | /IPR000047_Helix-turn-helix motif, lambda-like repressor /IPR001356_Homeobox /IPR009057_Homeodomain-like /IPR012287_Homeodomain-related |
|  | Msc | musculin | **/transcription /transcription,_DNA-dependent** **/regulation_of_transcription,_DNA-dependent** /regulation_of_cellular_metabolism **/regulation_of_transcription** | /DNA_binding **/transcription_regulator_activity** | /nucleus | **/MP:0000111_cleft palate /MP:0000729_abnormal myogenesis** /MP:0002058_neonatal lethality /MP:0003924_herniated diaphragm **/MP:0004234_abnormal masticatory muscle morphology /MP:0004236_absent masseter muscle /MP:0004237_abnormal pterygoid muscle morphology /MP:0004238_absent pterygoid muscle /MP:0004240_absent temporalis muscle /MP:0005369_muscle phenotype** |  | /IPR001092_Basic helix-loop-helix dimerisation region bHLH /IPR011598_Helix-loop-helix DNA-binding |
|  | Myod1 | **myogenic differentiation 1** | **/transcription /transcription,_DNA-dependent /regulation_of_transcription,_DNA-dependent** /development **/muscle_development /myoblast_cell_fate_determination /striated_muscle_development** /cell_differentiation /regulation_of_cellular_metabolism **/myoblast_differentiation /regulation_of_transcription** | /DNA_binding **/transcription_factor_activity /RNA_polymerase_II_transcription_factor_activity,_enhancer_binding** /protein_binding **/transcription_regulator_activity** | /nucleus | **/MP:0000729_abnormal myogenesis /MP:0000733_abnormal muscle development /MP:0000759_abnormal skeletal muscle morphology** |  | /IPR001092_Basic helix-loop-helix dimerisation region bHLH **/IPR002546_Myogenic basic muscle-specific protein** /IPR011598_Helix-loop-helix DNA-binding |
|  | Myog | myogenin | **/transcription /transcription,_DNA-dependent /regulation_of_transcription,_DNA-dependent /development /muscle_development /striated_muscle_development** /cell_differentiation /regulation_of_cellular_metabolism **/regulation_of_transcription** | /DNA_binding **/transcription_factor_activity /RNA_polymerase_II_transcription_factor_activity,_enhancer_binding /transcription_regulator_activity** | /nucleus | /MP:0000005_increased brown fat amount /MP:0000061_fragile skeleton /MP:0000150_abnormal rib morphology /MP:0000157_abnormal sternum morphology /MP:0000160_kyphosis  **/MP:0000729_abnormal myogenesis /MP:0000733_abnormal muscle development /MP:0000734_muscle hypoplasia /MP:0000759_abnormal skeletal muscle morphology /MP:0000761_thin diaphragm muscle** /MP:0001176_abnormal lung development /MP:0001178_pulmonary hypoplasia /MP:0001262_decreased body weight /MP:0001265_reduced body size /MP:0001404_no spontaneous movement /MP:0001491_unresponsive to tactile stimuli /MP:0001575_cyanosis /MP:0001785_edema /MP:0001953_respiratory failure /MP:0001961_abnormal reflex /MP:0002058_neonatal lethality **/MP:0002263_abnormal laryngeal muscle morphology /MP:0003084_abnormal skeletal muscle fiber morphology /MP:0005369_muscle phenotype** /MP:0005380_embryogenesis phenotype |  | /IPR001092_Basic helix-loop-helix dimerisation region bHLH **/IPR002546_Myogenic basic muscle-specific protein** /IPR011598_Helix-loop-helix DNA-binding |
|  | Pitx3 | paired-like homeodomain transcription factor 3 | **/transcription /transcription,_DNA-dependent /regulation_of_transcription,_DNA-dependent** /development /midbrain_developmet/  regulation_of_cellular_metabolism /regulation_of_transcription **/neuron_development** | /DNA_binding **/transcription_factor_activity** /sequence-specific_DNA_binding | /nucleus **/transcription_factor_complex** | /MP:0000897_abnormal midbrain /MP:0001265_reduced body size /MP:0001297_microphthalmia /MP:0001299_abnormal eye distance or position /MP:0001317_abnormal pupil morphology /MP:0001325_abnormal retina morphology /MP:0001346_abnormal lacrimal gland morphology /MP:0001402_hypoactivity /MP:0001921_reduced fertility /MP:0002864_abnormal ocular fundus morphology /MP:0005102_abnormal iris pigmentation /MP:0005193_abnormal anterior eye segment morphology /MP:0005195_abnormal posterior eye segment morphology /MP:0005545_abnormal lens development |  | /IPR001356_Homeobox /IPR003654_Paired-like homeodomain protein, OAR /IPR009057_Homeodomain-like /IPR012287_Homeodomain-related |
|  | Rxrg | retinoid X receptor gamma | **/transcription /transcription,_DNA-dependent /regulation_of_transcription,_DNA-dependent /regulation_of_transcription** | /DNA_binding **/transcription_factor_activity** /steroid_hormone_receptor_activity /signal_transducer_activity/receptor_activity /ligand-dependent_nuclear_receptor_activity /transmembrane_receptor_activity /steroid_binding /zinc_ion_binding /sequence-specific_DNA_binding /metal_ion_binding | /nucleus |  | /Adipocytokine signaling pathway | /IPR000003_Retinoid X receptor /IPR000536_Nuclear hormone receptor, ligand-binding /IPR001628_Nuclear hormone receptor, DNA-binding /IPR001723_Steroid hormone receptor /IPR008946_Nuclear receptor, ligand-binding |
|  | Zim1* | zinc finger, imprinted 1 | **/transcription /transcription,_DNA-dependent /regulation_of_transcription,_DNA-dependent /regulation_of_transcription** | /nucleic_acid_binding /zinc_ion_binding /metal_ion_binding | /intracellular /nucleus |  |  | /IPR001909_KRAB box /IPR007086_Zinc finger, C2H2-subtype /IPR007087_Zinc finger, C2H2-type |

**Table S2: Annotation terms associated with those nodes added via the Logit asserted edges.**

The table highlights informative annotation evidence (in **bold**) of those nodes added through the incorporation of Logit edges into the network. InterPro , GO Biological Process (GO_BP), Molecular Function (GO_MF) and Cellular Component (GO_CC) annotation terms, KEGG pathway information, and associated phenotypes (extracted from MGI) are shown here. The far left column indicates the functional cluster the group of nodes were attributed to. An * indicates those nodes which were subject to further biological evaluation and validation.
